# Supplementary material for: High triplet energy host material with a 1,3,5-oxadiazine core from a one-step interrupted Fischer indolization
Source: Commun Chem. 2024 Dec 19;7:298. doi: 10.1038/s42004-024-01377-y (PMC11659277; doi:10.1038/s42004-024-01377-y)
Supplement: Supplementary file 2 — Supplementary Material [file 42004_2024_1377_MOESM2_ESM.pdf]

Supplementary Information for:

High Triplet Energy Host Material with 1,3,5-Oxadiazine Core from a One-step Interrupted Fischer Indolization

Charlotte Riley,[1] Hwan-Hee Cho,[2] Nguyen Le Phuoc,[3] Mikko Linnolahti,[3]* Neil C. Greenham,[2]* and Alexander S. Romanov[2]*

[1] Department of Chemistry, The University of Manchester, Oxford Rd, Manchester M13 9PL, United Kingdom

[2] Department of Physics, Cavendish Laboratory, Cambridge University CB3 0HF, United Kingdom

[3] Department of Chemistry University of Eastern Finland, FI-80101 Joensuu, Finland

Supplementary Methods

1. Materials and Methods 2
2. Molecular Synthesis, Characterisation and Mechanism 2
3. Single Crystal Diffraction Analysis 10
4. Electrochemical and Thermal Properties 13
5. Photophysical Characterisation 14
6. Device Fabrication and Characterisation 26
7. Computational Details 26
8. Optimised coordinates 34
9. Supplementary References 39

1. Materials and Methods

All solvents and reactants required for synthesis are commercially available and were used as received. All reactions were performed under ambient conditions. ^{1}H, ^{13}C, and ^{19}F NMR spectra were recorded on Bruker AVIII HD 400 MHz or AV II 700 MHz spectrometer. ^{1}H and ^{13}C NMR spectra were referenced to CD_3Cl at δ 7.27 (^{13}C δ 77.16). Mass spectrometry data was obtained on a Thermo Orbitrap Exactive Plus Extended Mass Range Spectrometer using an APCI(ASAP) probe by the Mass Spectrometry Laboratory at the University of Manchester. All electrochemical experiments were performed using an Autolab PGSTAT 302N computer-controlled potentiostat. Cyclic voltammetry (CV) was performed using a three-electrode configuration consisting of a glassy carbon macrodisk working electrode (GCE) (diameter of 3 mm; BASi, Indiana, U.S.A.) combined with a Pt wire counter electrode (99.99%; GoodFellow, Cambridge, U.K.) and an Ag wire pseudoreference electrode (99.99%; GoodFellow, Cambridge, U.K.). The GCE was polished between experiments using alumina slurry (0.3 µm), rinsed in distilled water and subjected to brief sonication to remove any adhering alumina microparticles. The metal electrodes were then dried in an oven at 100 °C to remove residual traces of water, the GCE was left to air dry and residual traces of water were removed under vacuum. The Ag wire pseudoreference electrodes were calibrated to the ferrocene/ferrocenium couple in 1,2-difluorobenzene (DFB) at the end of each run to allow for any drift in potential, following IUPAC recommendations.[1] All electrochemical measurements were performed at ambient temperatures under an inert N_2 atmosphere in 1,2-difluorobenzene (DFB) containing the complex under study (0.14 mM) and the supporting electrolyte [*n*-Bu_4N][PF_6] (0.13 M). Data were recorded with Autolab NOVA software (v. 1.11). Thermogravimetric analysis was performed with a TA Instruments SDT650 simultaneous thermal analyser under a stream of nitrogen.

2. Synthesis, Characterisation and Mechanism

The desired 1,3,5-oxadiazine compounds were synthesised using the following general procedure:

Cyclohexane carboxaldehyde (3 equiv, 31.12 mmol) was added slowly to a solution of the substituted phenyl hydriazine hydrochloride (2 equiv, 20.75 mmol) in glacial acetic acid (120 mL). The resulting mixture was stirred at 60 °C for 3 hours, a colour change from orange to dark red was observed. The reaction mixture was concentrated under reduced pressure and then neutralised with K_2CO_3 solution. The product was extracted into DCM (3 × 60 mL) and the organic layer dried with anhydrous $MgSO_4$ before filtering through a silica plug (10 cm).

Removal of the solvent under reduced pressure yielded the crude product as a white solid suspended in yellow oil. The pure product was obtained was a white powder by washing with cold pentane.

NON

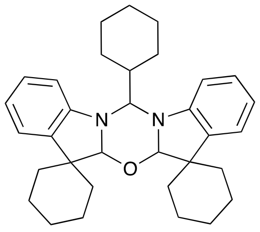

Yield: 26%

^{1}H NMR (500 MHz, $CDCl_3$): δ 7.00 (t, *J = 6.9 Hz*, 2H, H_b) 6.91 (d, *J = 6.8 Hz*, 2H, H_d) 6.62 (t, *J= 7.5 Hz*, 4H, H_a H_c) 5.23 (s, 2H, H_e) 4.95 (d, *J = 10.6 Hz*, 1H, H_f) 2.27 (m, 1H, H_g) 2.07-1.09 (m, 30H, CH, cyclohexyl rings overlapped).

^{13}C{^{1}H} NMR (75 MHz, $CDCl_3$) : δ 148.9 (CH, C_1) 137.7 (CH, C_6) 127.4 (CH, C_3) 122.4 (CH, C_5) 118.2 (CH, C_4) 107.2 (CH, C_2) 91.2 (CH, C_8) 68.7 (CH, C_9) 48.6 (C, C_7) 39.2 (CH, C_{10}) 35.6 (CH_2, cyclohexyl), 30.1 (CH_2, cyclohexyl) 29.0 (CH_2, cyclohexyl) 26.0 (CH_2, cyclohexyl) 26.7(CH_2, cyclohexyl) 25.9(CH_2, cyclohexyl) 24.1(CH_2, cyclohexyl) 23.6(CH_2, cyclohexyl)

HRMS $C_{33}H_{42}N_2O$ theoretical 483.3370 MS (APCI 483.3387)

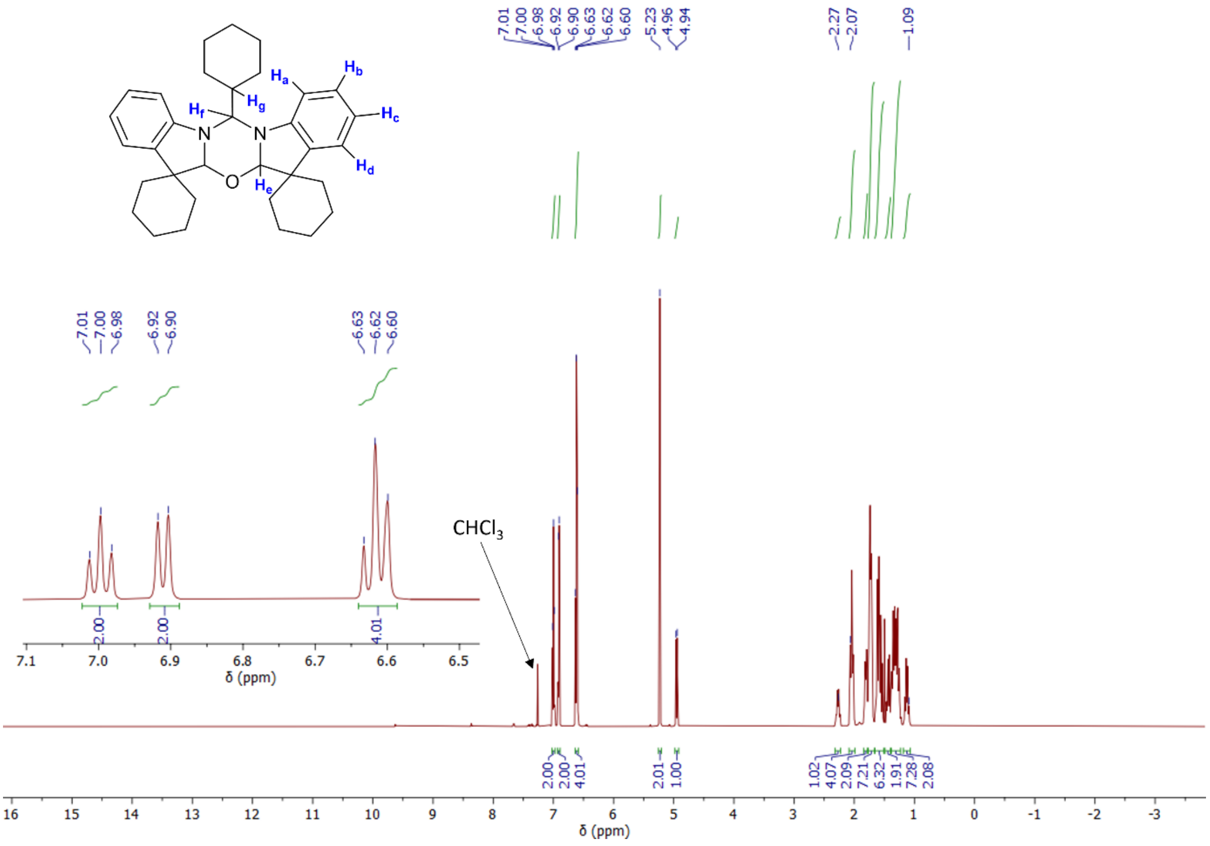

Figure S1. ^{1}H NMR (500 MHz, $CDCl_3$) of **NON**

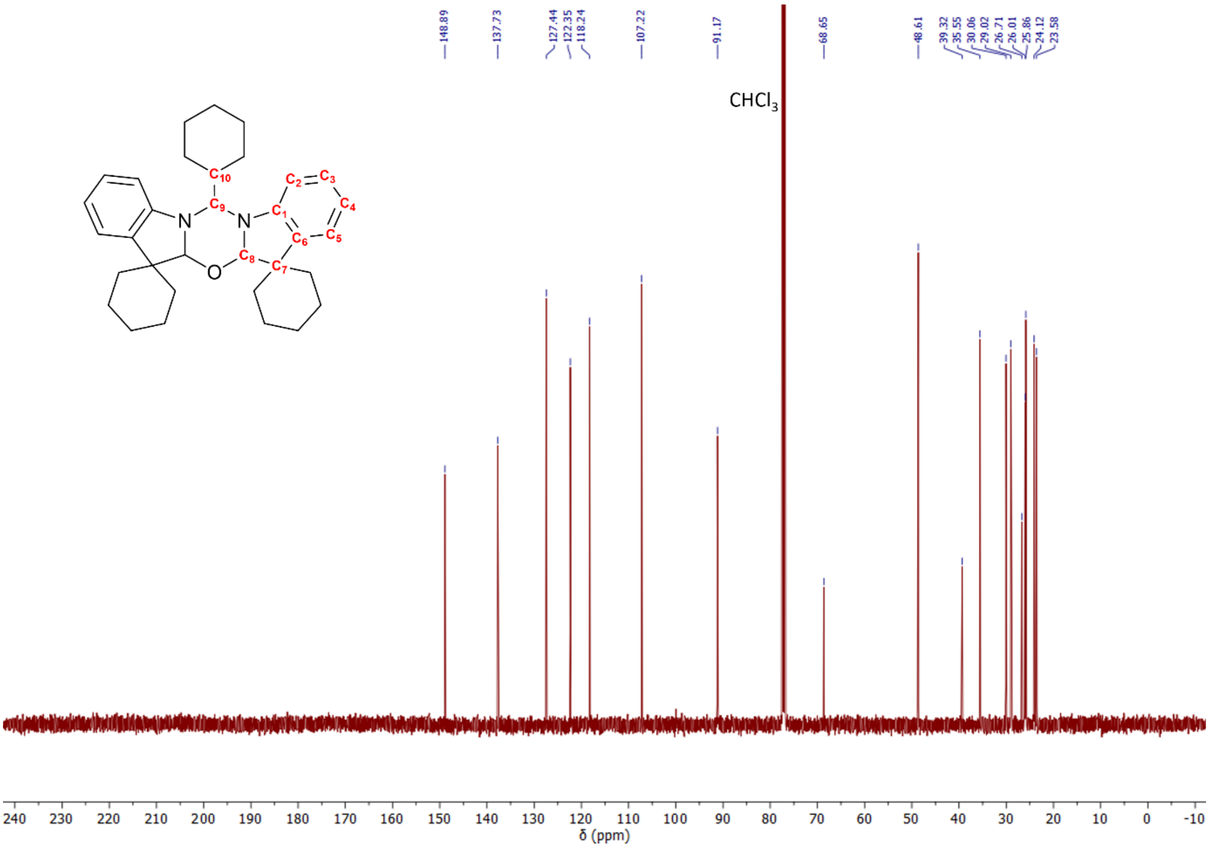

Figure S2. $^{13}C\{^{1}H\}$ NMR (75 MHz, $CDCl_3$) of **NON**

NON-CF_3

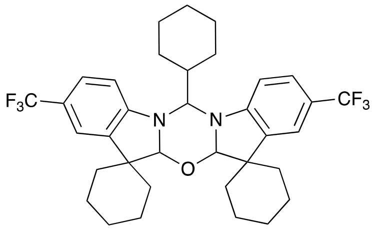

Yield: 82%

^{1}H NMR (500 MHz, $CDCl_3$): δ 7.28 (d, *J=8.3 Hz*, 2H, H_b) 7.13 (s, 2H, H_c) 6.60 (d, *J= 8.4 Hz*, 2H, H_a) 5.29 (s, 2H, H_d) 4.98 (d, *J = 10.9 Hz*, 1H, H_e) 2.28 (m, 1H, H_f) 2.08-0.87 (m, 30H, CH_2 cyclohexyl rings overlapped).

$^{13}C\{^{1}H\}$ NMR (75 MHz, $CDCl_3$): δ 151.3 (CH, C_1) 138.3 (CH, C_6) 126.1 (CH, q, $^{3}J_{CF} = 3.9$ *Hz*, C_5) 125.4 (CF, broad q, $^{1}J_{CF}= 275$ *Hz*, C_{11}) 120.8 (CH, q, $^{2}J_{CF}=32$ *Hz*, C_4) 119.9 (CH, q, $^{3}J_{CF}= 3.7$ *Hz*, C_3) 106.5 (CH,C_2) 91.50 (CH, C_8) 69.2(CH, C_9) 48.7 (CH,C_7) 39.0 (CH, C_{10}) 35.5 (CH_2, cyclohexyl), 31.7 (CH_2, cyclohexyl) 29.9 (CH_2, cyclohexyl) 28.9 (CH_2, cyclohexyl) 26.5 (CH_2, cyclohexyl) 25.9 (CH_2, cyclohexyl) 25.6 (CH_2, cyclohexyl) 24.0 (CH_2, cyclohexyl) 23.4(CH_2, cyclohexyl) 22.8(CH_2, cyclohexyl)

^{19}F NMR (470 MHz): -60.7 (CF_3-aromatic)

HRMS $C_{33}H_{42}F_6N_2O$ theoretical 618.3039 MS (APCI 618.3056)

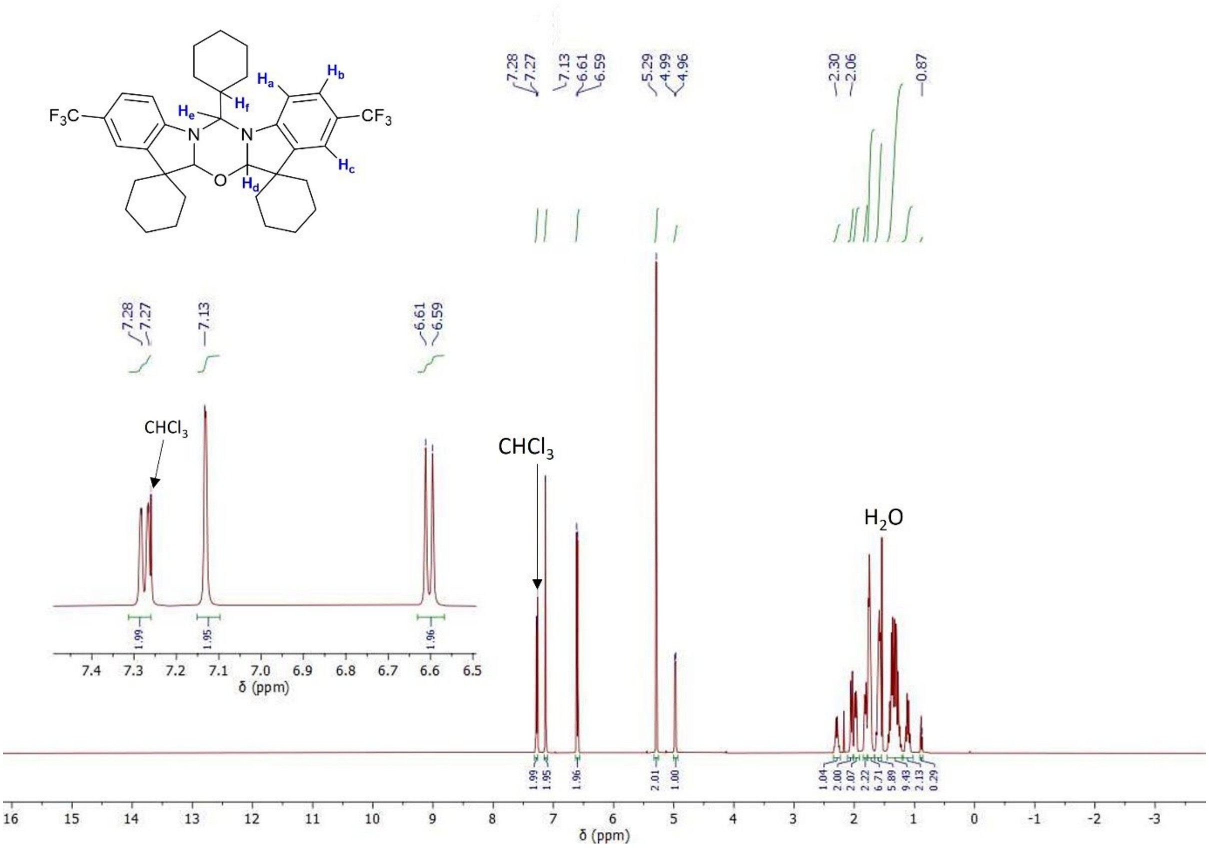

Figure S3. ^{1}H NMR (500 MHz, $CDCl_3$) of **NON-CF$_3$**

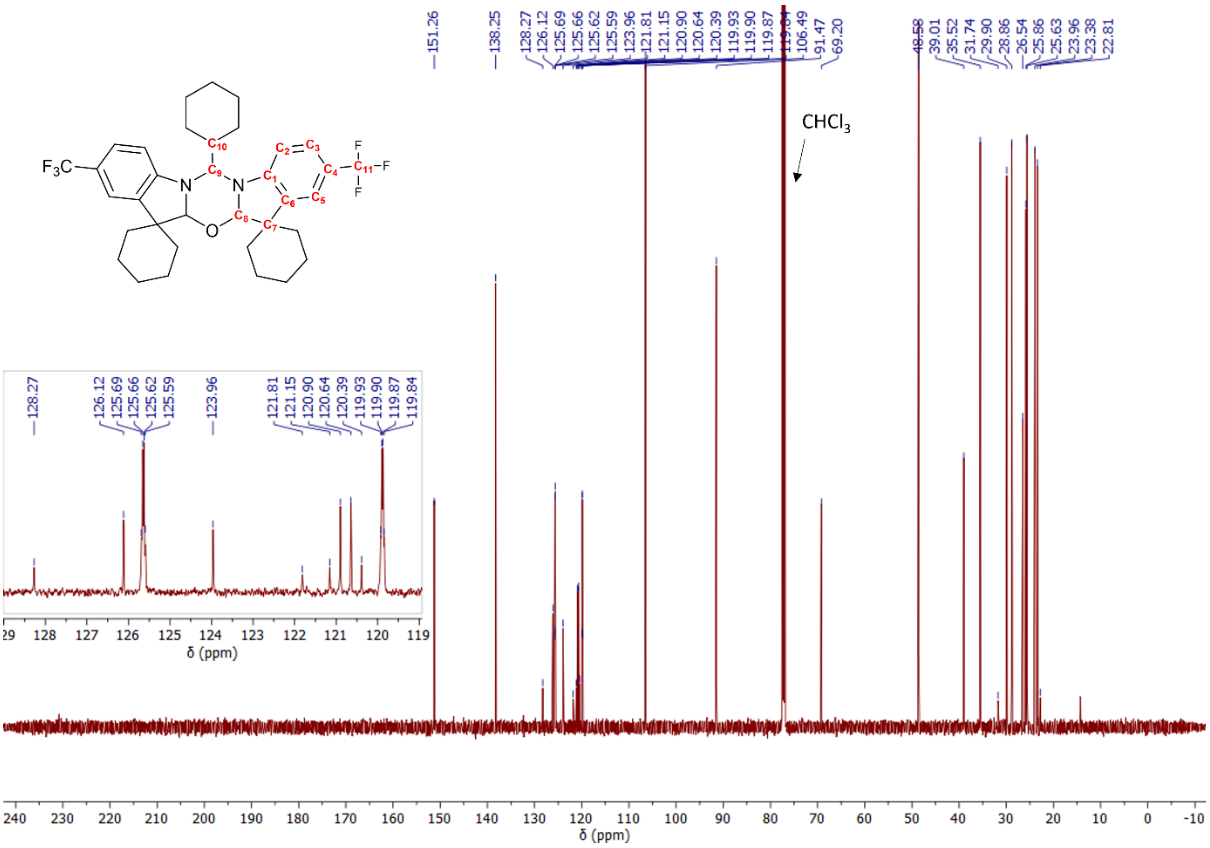

Figure S4. $^{13}C\{^{1}H\}$ NMR (75 MHz, $CDCl_3$) of **NON-CF$_3$**

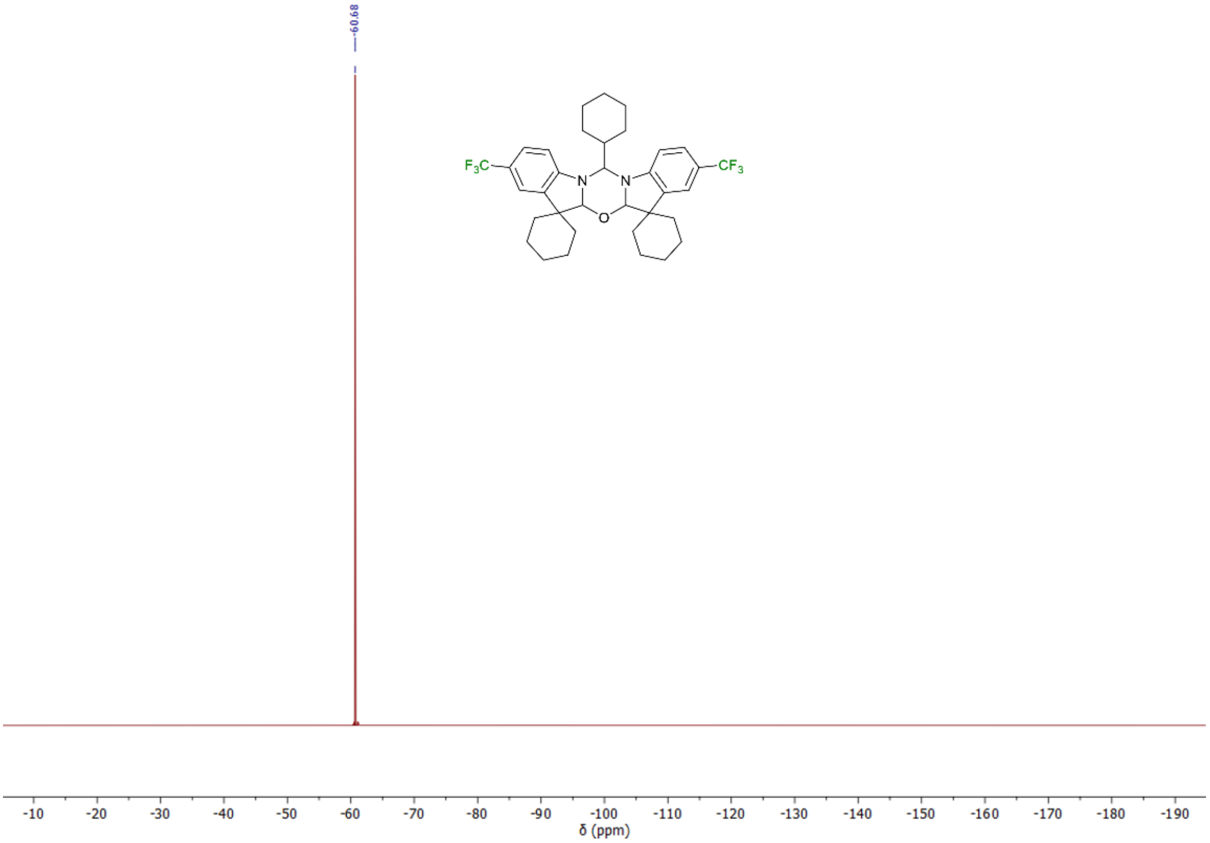

Figure S5. ^{19}F NMR (470 MHz) of **NON-CF$_3$**

NON-Br

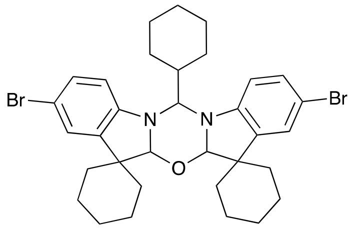

Yield: 22%

^{1}H NMR (500 MHz, $CDCl_3$): δ 7.07 (d, *J= 8.5 Hz*, 2H, H_b) 6.98 (s, 2H, H_c) 6.45 (d, *J=8.3* , 2H, H_a) 5.19 (s, 2H, H_d) 4.80 (d, *J = 10.7 Hz*, 1H, H_e) 2.22 (m, 1H, H_f) 2.02-0.89 (m, ~30H, CH_2, cyclohexyl rings overlapped).

^{13}C{^{1}H} NMR (75 MHz, $CDCl_3$) 147.8 (CH, C_1) 140.2 (CH, C_6) 130.0 (CH, C_3) 125.8 (CH, C_5) 110.2 (CH, C_2) 108.8 (C-Br, C_4) 91.1 (CH, C_8) 69.1 (CH, C_9) 48.9 (C,C_7) 38.9 (CH, C_{10}) 35.3 (CH_2, cyclohexyl), 31.7 (CH_2, cyclohexyl) 30.1 (CH_2, cyclohexyl) 28.8 (CH_2, cyclohexyl) 26.6 (CH_2, cyclohexyl) 25.9 (CH_2, cyclohexyl) 25.7 (CH_2, cyclohexyl) 23.9 (CH_2, cyclohexyl) 23.5 22.8 (CH_2, cyclohexyl) (CH_2, cyclohexyl)

HRMS $C_{33}H_{40}Br_2N_2O$ 638.1532 MS (APCI 638.1502)

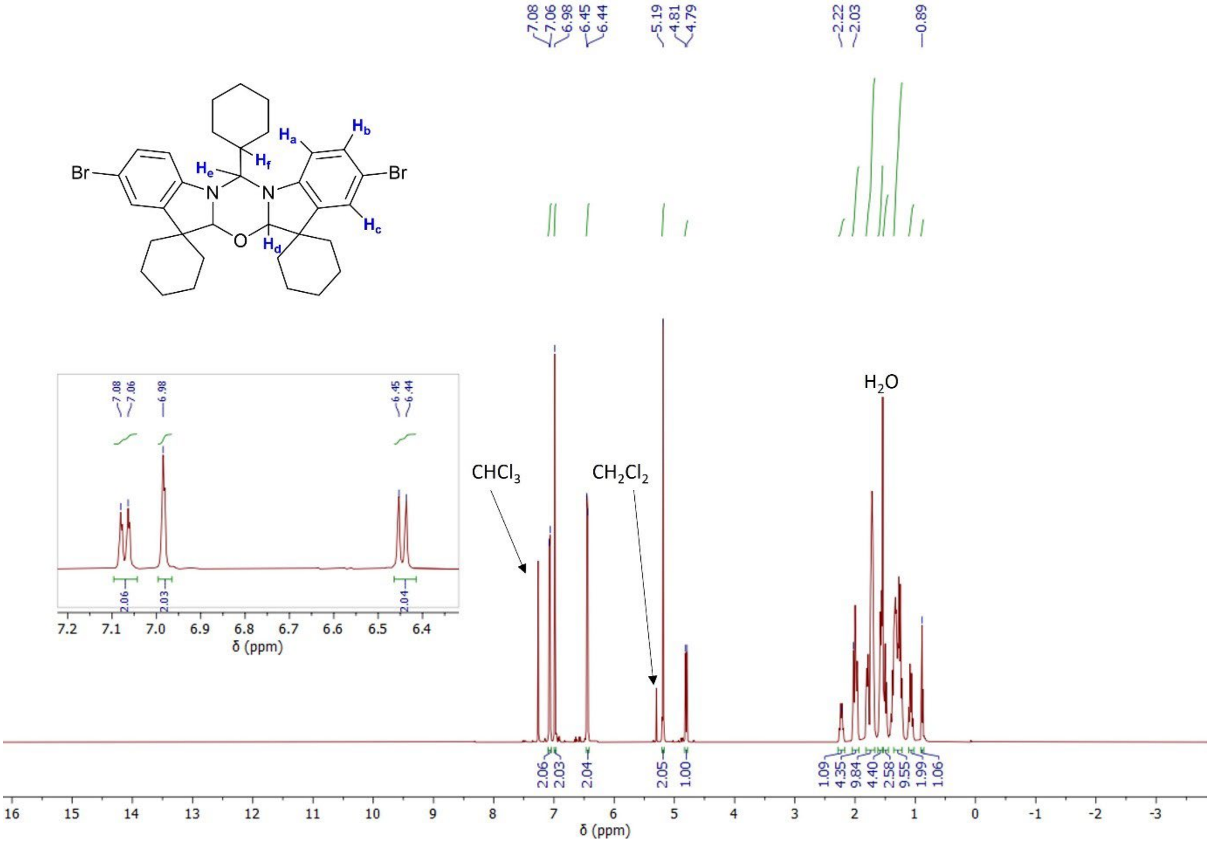

Figure S6. ^1H NMR (500 MHz, $CDCl_3$) of **NON-Br**

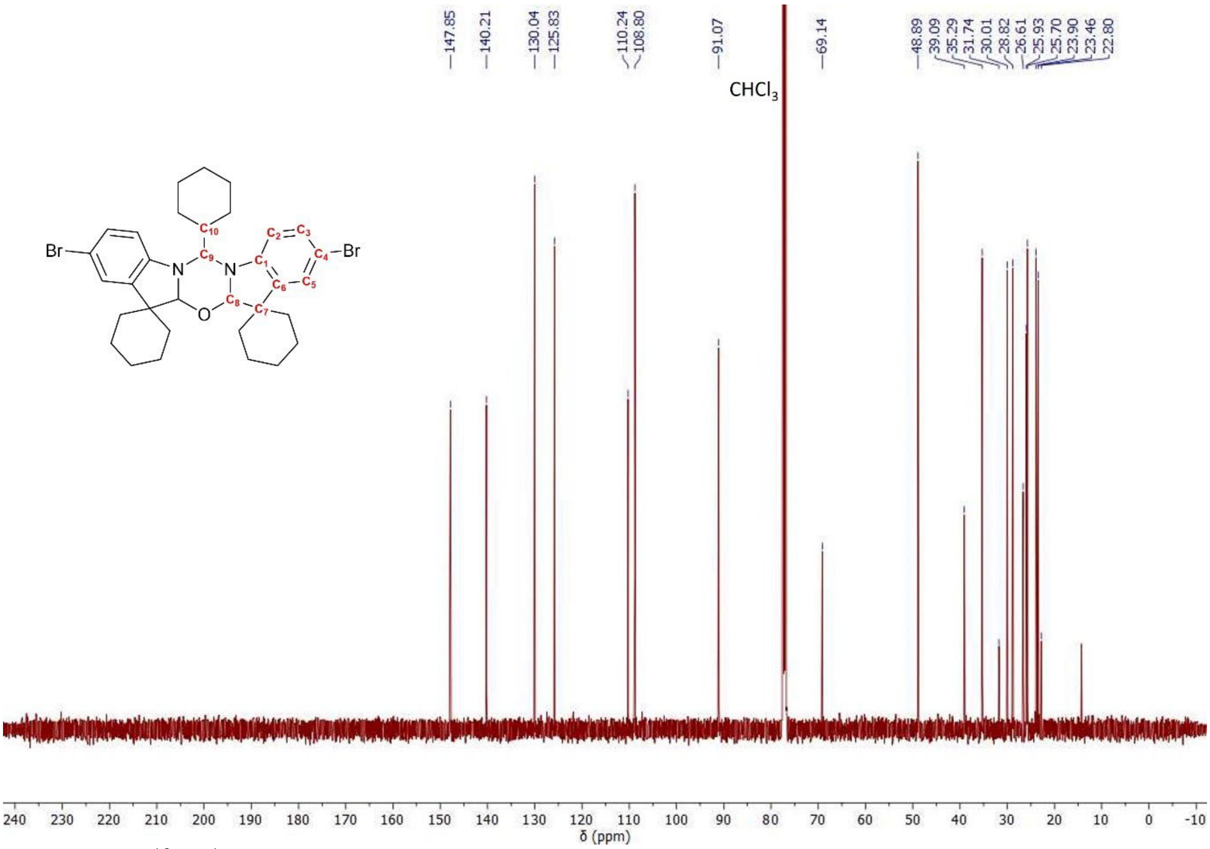

Figure S7. $^{13}C\{^{1}H\}$ NMR (75 MHz, $CDCl_3$) of **NON-Br**

Mechanism for 1,3,5-oxadiazine formation

Whilst detailed mechanistic studies fall outside the scope of this work we have provided a potential curly arrow mechanism for the formation of NON compounds.

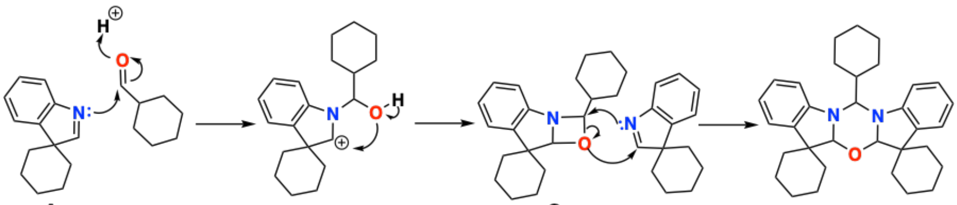

Scheme S1: Curly arrow mechanism for the formation of NON

The reaction proceeds with the formation of the classical fisher product (**A**). The nitrogen electron lone pair of **A** is then available to attach the carbonyl bond of the carboxaldehyde which is added to the reaction mixture in excess giving suggested intermediate **B**. In a manner analogous to the interrupted Fischer indole mechanism intramolecular nucleophilic attack within **B** leads to the formation of **C**, a highly strained four-membered ring intermediate. A final equivalent of **A** initiates a ring opening reaction to yield the observed 1,3,5-oxadiazine.

Attempts to isolate any additional products from the reaction were unsuccessful, indicating any imine which formed was consumed.

3. Crystallographic data

Crystals suitable for X-ray diffraction study were obtained by slow evaporation of the hexane solution at room temperature. Crystals were mounted in oil on glass fiber and fixed on the diffractometer in a cold nitrogen stream. Data was collected using four-cycle SuperNova, Single source (Kα Mo) X-ray Source, Oxford Rigaku diffractometer at 100 K. Data were processed using the CrystAlisPro-CCD and –RED software.[2] Multi-scan absorption correction was applied for all crystals. Structures were solved by direct method/intrinsic phasing and refined by the full-matrix least-squares against F^2 in an anisotropic (for non-hydrogen atoms) approximation. All hydrogen atoms were positioned geometrically and constrained to ride on their parent atoms with C-H = 0.95-1.00 Å, and U_{iso} = 1.2−1.5 U_{eq} (parent atom). All calculations were performed using the SHELXL software[3] and Olex2 graphical user interface.[4]

Table S1. Summary of crystallographic data and structure refinement

	NON	NON-CF3	NON-Br
CCDC №	2363678	2363679	2363680
Empirical formula	$C_{36}H_{49}N_2O$	$C_{35}H_{40}F_6N_2O$	$C_{33}H_{40}Br_2N_2O$
Molecular weight	525.77	618.69	640.49
Crystal system	Triclinic	Monoclinic	Monoclinic
Space group	*P*-1	$P2_1/n$	$P2_1/n$
Crystal colour, habit	None, none, none	None, none, none	None, none, none
Crystal size (mm^3)	0.37× 0.29 × 0.25	0.32 × 0.25 × 0.23	0.4 × 0.37 × 0.30
a (Å)	10.3980(4)	10.1399(4)	10.4033(3)
b (Å)	10.5761(8)	15.4499(6)	14.9493(4)
c (Å)	16.6417(11)	19.9660(7)	18.9504(5)
(°)	79.594(6)	90	90
(°)	81.659(4)	101.603(4)	104.082(3)
(°)	64.055(6)	90	90
V (Å^3)	1613.83(19)	3012.9(2)	2858.64(14)
Z	2	4	4
Dcalc (g cm^{-3})	1.082	1.364	1.488
2θmax (°)	55.99	58.15	58.31

Abs. coeff., (Mo-K) (mm^{-1})	0.064	0.107	2.865
T (K)	100.00	100.15	100.00
Number of collected reflections	22492	25412	24700
Number of independent reflections	7442	7250	6819
R_{int}	0.0821	0.0482	0.0422
Number of parameters	351	397	343
R_1 (on F for observed reflexions)	0.1061	0.0506	0.0367
wR_2 (on F2 for all reflexions)	0.3517	0.1282	0.0821
F(000)	574.0	1304.0	1320
Goodness-of-fit	1.042	1.042	1.019

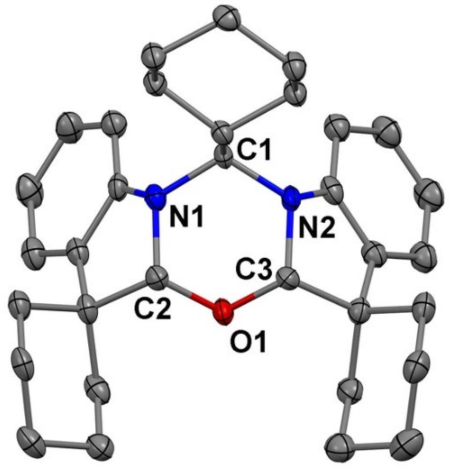

Figure S8. X-ray crystal structure of **NON**. Ellipsoids are shown at the 50% level. Hydrogen atoms emitted for clarity.

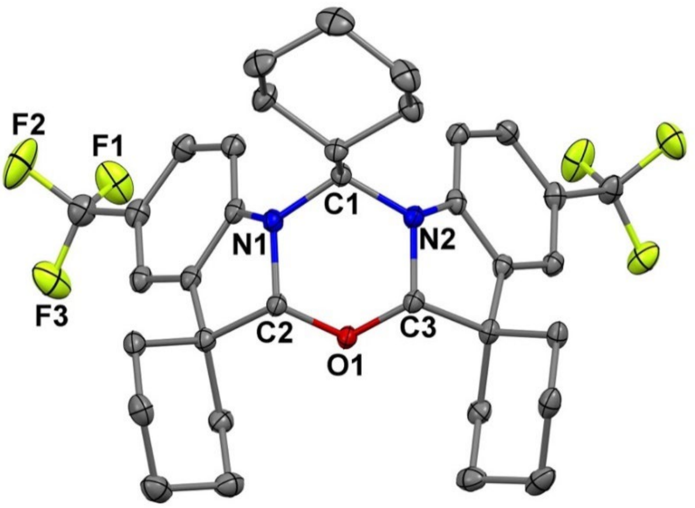

Figure S9. X-ray crystal structure of **NON-CF_3**. Ellipsoids are shown at the 50% level. Hydrogen atoms emitted for clarity.

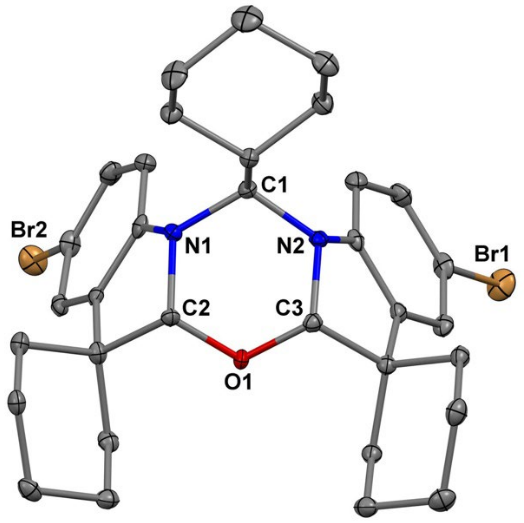

Figure S10. X-ray crystal structure of **NON-Br**. Ellipsoids are shown at the 50% level. Hydrogen atoms emitted for clarity.

4. Electrochemical and Thermal Properties

Electrochemical analysis was conducted for complexes **NON**, **NON-CF$_3$** and, **NON-Br** using a glassy carbon electrode in 1,2-difluorobenzene (DFB) solution (1.4 mM) with [n-Bu$_4$]PF$_6$ as the supporting electrolyte (0.13 M), scan rate 0.1 Vs^{-1}

Table S2: Formal electrode potentials (peak position E_p for irreversible and $E_{1/2}$ for quasi-reversible processes (*), *V, vs.* FeCp2), onset potentials (*E, V, vs.* FeCp2), peak to peak separation in parentheses for quasi reversible processes (ΔE_p in mV), and E_{LUMO} (eV) compounds **NON**, **NON-CF$_3$** and, **NON-Br**. [a]

Compound	Oxidation			E_{HOMO}
	E_{1st}	$E_{onset\ ox}$	E_{2nd}	eV
**NON**	+0.50	+0.37	-	– 5.76
**NON-CF$_3$**	+0.90 (100)	+0.75	+1.38	– 6.14
**NON-Br**	+0.62 (90)	+0.51	+1.12 (140)	– 5.90

[a] In DFB solution, recorded using a glassy carbon electrode, concentration 1.4 m*M*, supporting electrolyte [*n*Bu$_4$N][PF$_6$] (0.13 *M*), measured at 0.1 V s-1. $E_{HOMO} = -(E_{onset\ ox\ Fc/Fc+} + 5.39)\ eV$ (*Adv. Mater.* **2011**, *23*, 2367-2371).

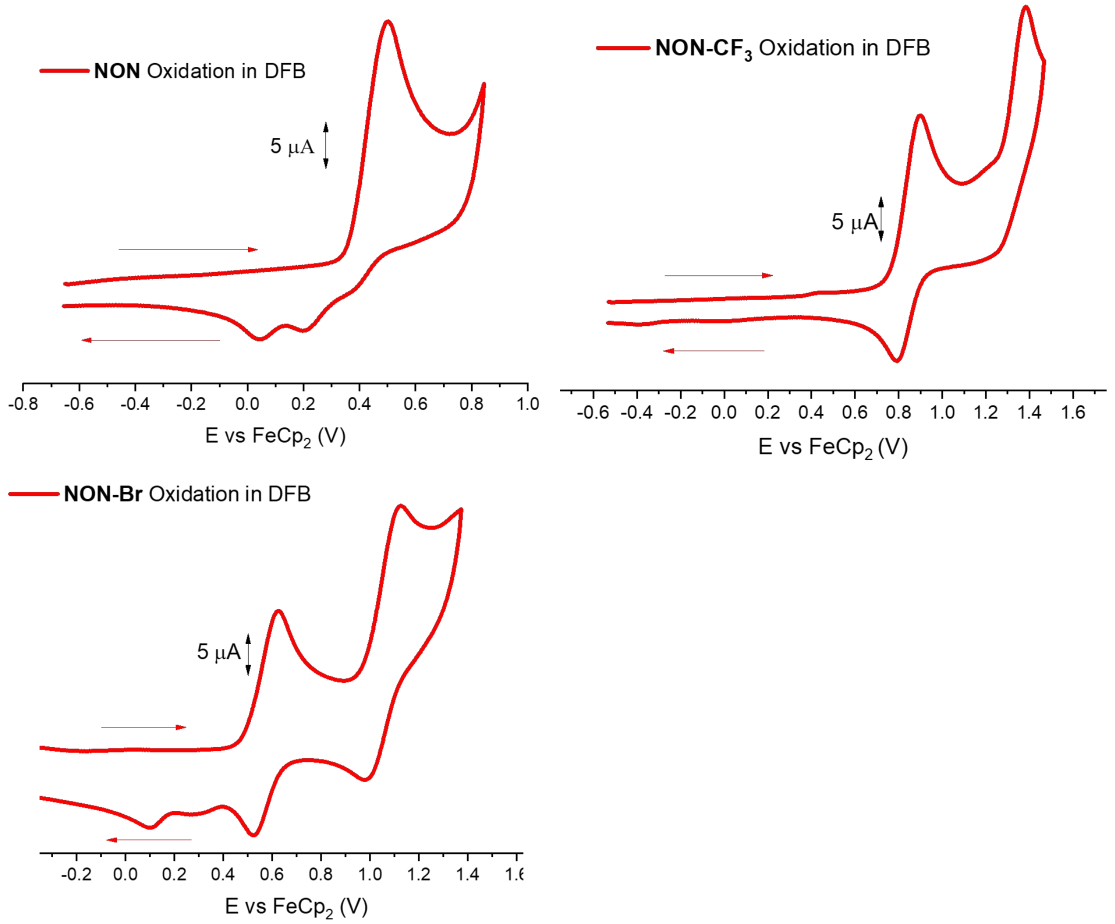

Figure S11: Oxidation only cyclic voltammogram for **NON**, **NON-CF$_3$**, and **NON-Br**. Recorded using a glassy carbon electrode in DFB solution (1.4 mM) with [n-Bu$_4$N]PF$_6$ as supporting electrolyte (0.13 M), scan rate 0.1 V s-1.

Thermogravimetric Analysis

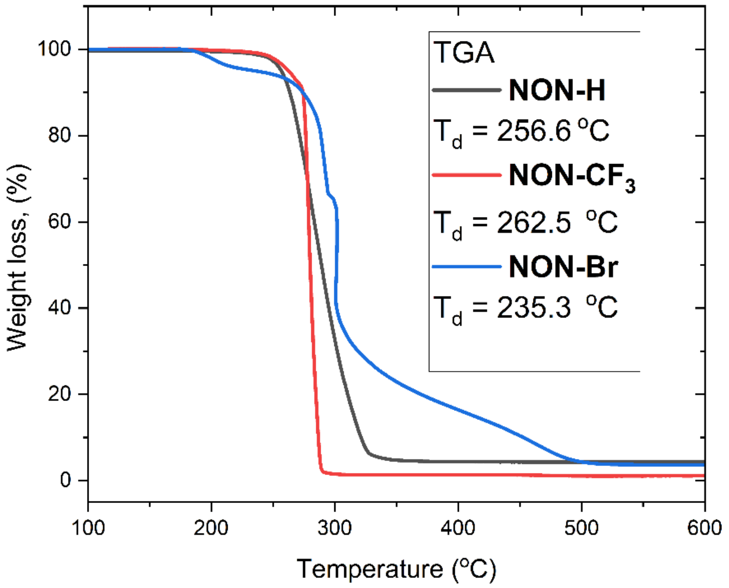

Figure S12: TGA curves for compounds NON, NON-CF_3 and NON-Br. Decomposition temperature (T_d) indicates temperature at 5% weight loss.

5. Photophysical Characterisation

Photoluminescence Quantum Yield

Quantum yields have been measured in air for solid samples and under nitrogen for solutions. Photoluminescence quantum yields were recorded using an Hamamatsu Quantaurus-QY C11347-11. Quantum yields have been measured in air for solid samples and under nitrogen for solutions.

Steady-state Photoluminescence

Steady-state PL spectra were recorded using an Edinburg Instruments FLS980 spectrofluorimeter. The light source was a monochromated Xenon arc lamp; excitation wavelength varied. Samples were measured in air or under flowing nitrogen, at room temperature.

UV-Vis Absorption

UV-Vis spectra were measured using a Varian Cary 5000 UV-Vis-NIR spectrometer and Shimadzu UV-3600 Plus UV-VIS-NIR spectrophotometer. The spectrometer has a PMT detector for wavelength ranges from UV to visible, as well as InGaAs and PbS detectors for NIR. The light source used was a deuterium lamp for wavelengths less than 280nm and a tungsten halogen lamp for higher wavelengths.

Table S3: UV-vis data for compounds **NON**, **NON-CF$_3$**, and **NON-Br** in cyclohexane, THF and CH_2Cl_2 solutions

Compound	λ_{abs} [nm], (10^3 ε/M^{-1} cm^{-1})		
	cyclohexane	THF	CH_2Cl_2
**NON**	293 (9.4), 303 (sh)[a]	260 (24), 293 (8.4), 303 (sh)	264 (28), 294 (10.5), 304 (sh)
**NON-CF$_3$**	267 (22.4), 302 (sh)	271 (17.8), 302 (sh)	273 (21.2), 302 (sh)
**NON-Br**	266 (18.6), 304 (4.9)	267 (11.4), 303 (3.4)	270 (15.7), 304 (4.1)

[a] sh = shoulder

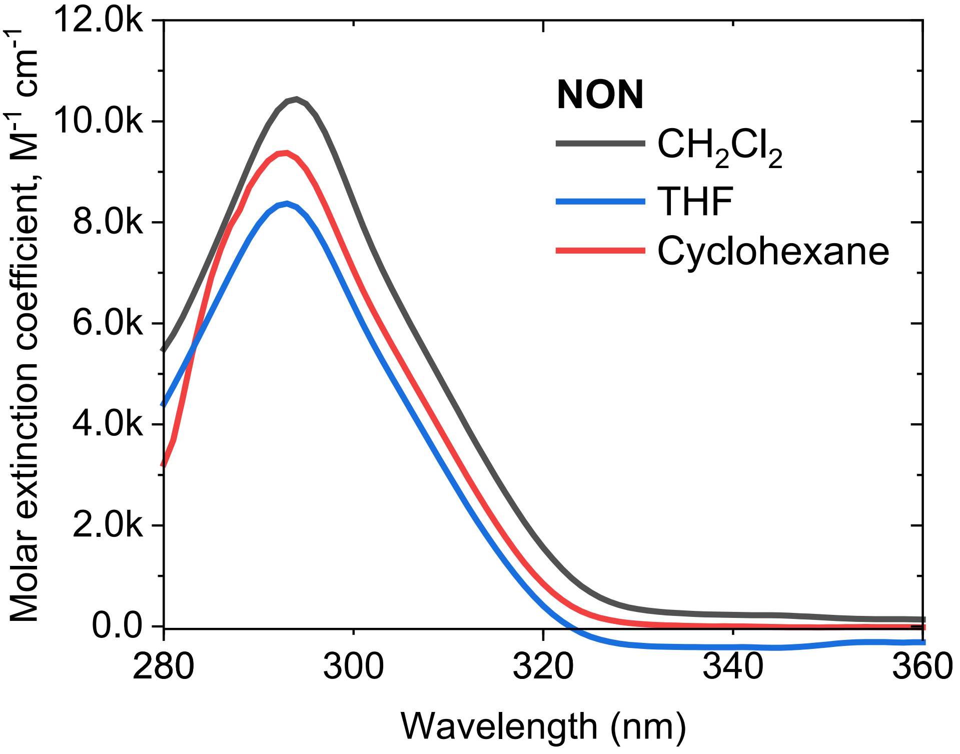

Figure S13: UV-vis absorption spectra for **NON** in CH_2Cl_2, THF and Toluene solution at 295 K

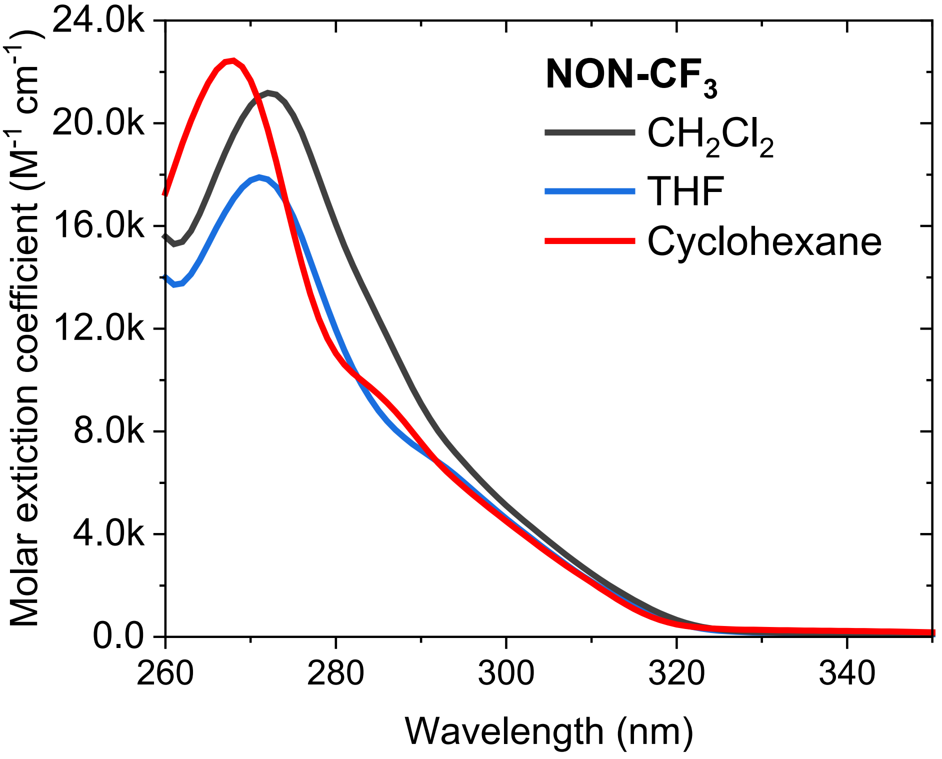

Figure S14: UV-vis absorption spectra for **NON-CF$_3$** in CH_2Cl_2, THF and cyclohexane solution at 295 K

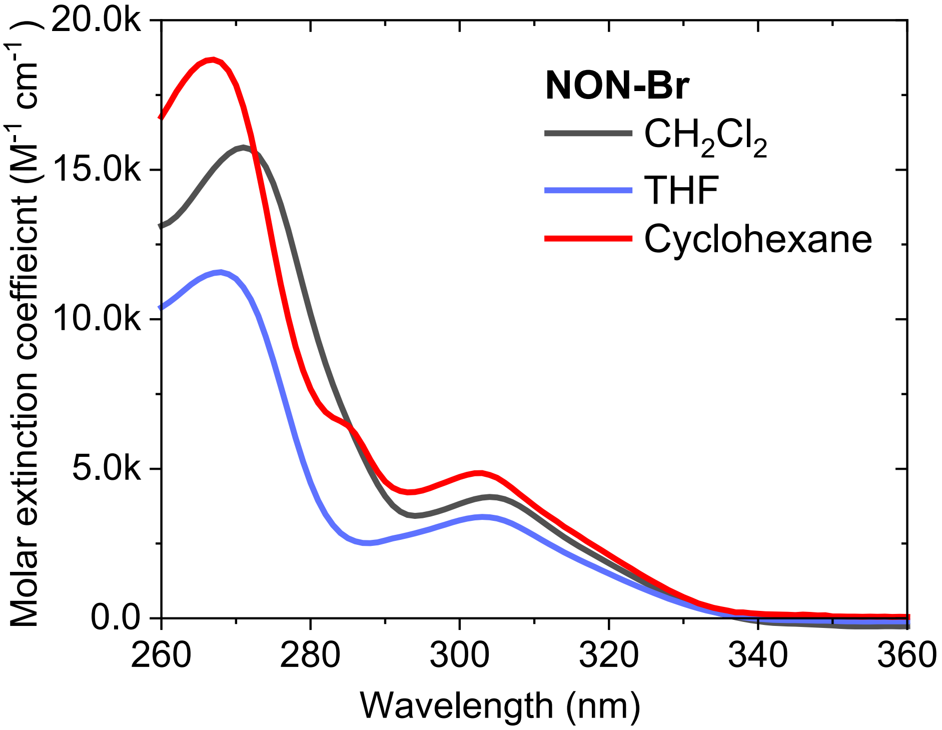

Figure S15: UV-vis absorption spectra for **NON-Br** in CH_2Cl_2, THF and cyclohexane solution at 295 K

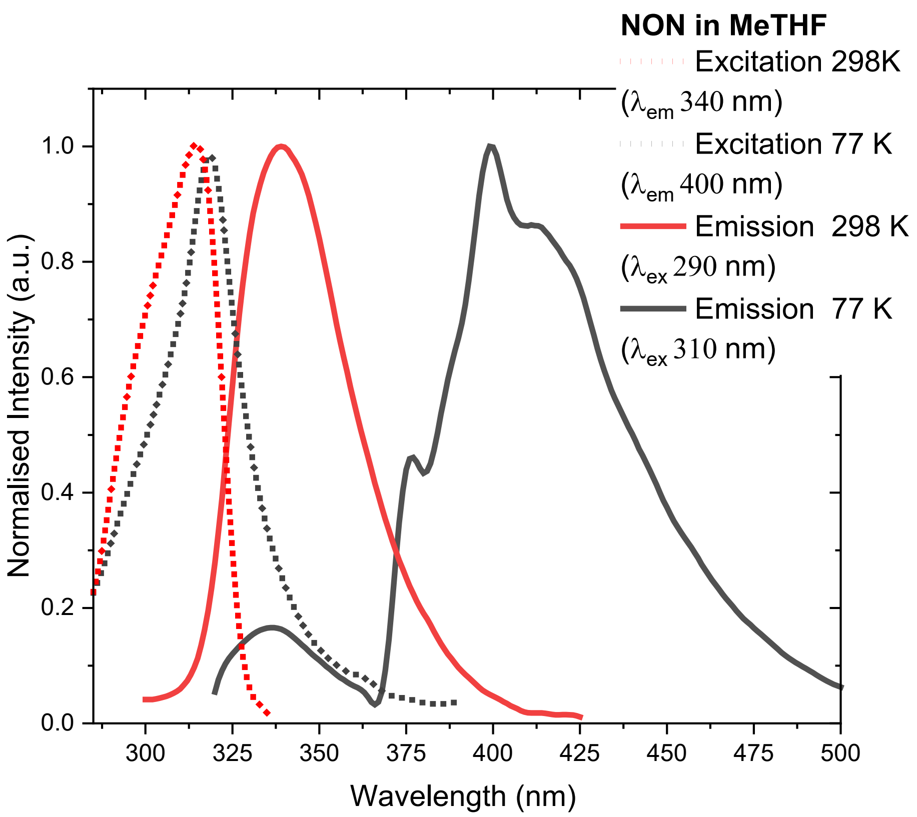

Figure S16: Excitation and Emission spectra for NON in MeTHF solution at 298 and 77 K

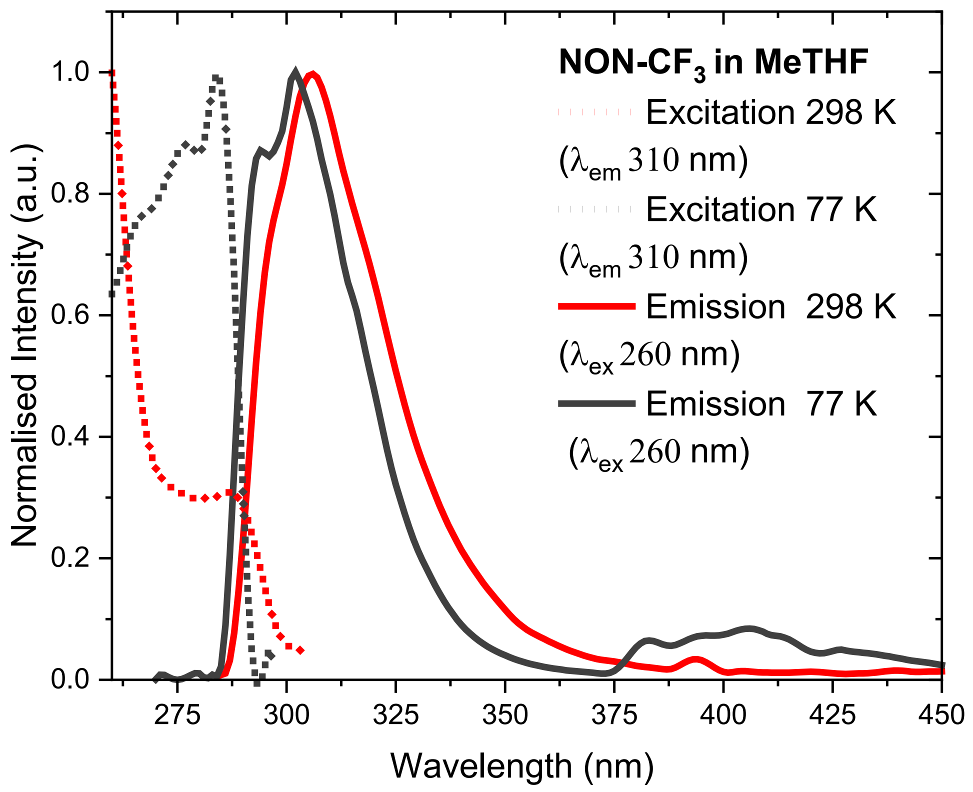

Figure S17: Excitation and Emission spectra for NON-CF_3 in MeTHF solution at 298 and 77 K

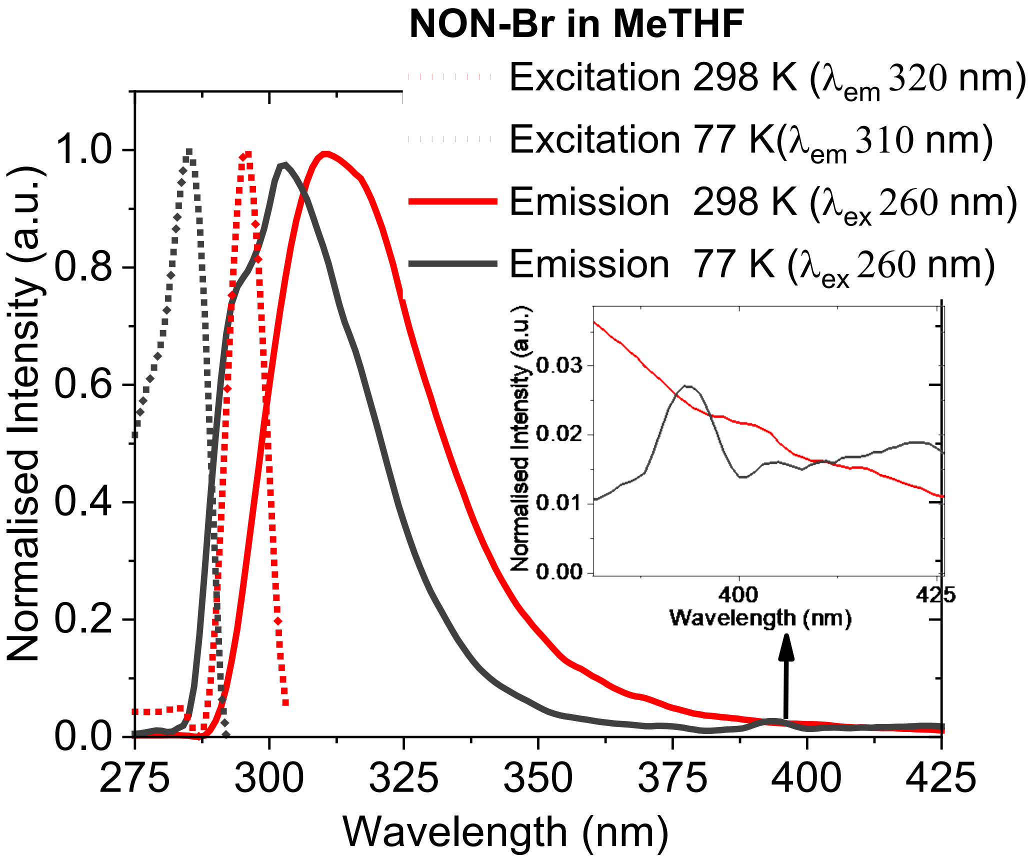

Figure S18: Excitation and Emission spectra for NON-Br in MeTHF solution at 298 and 77 K

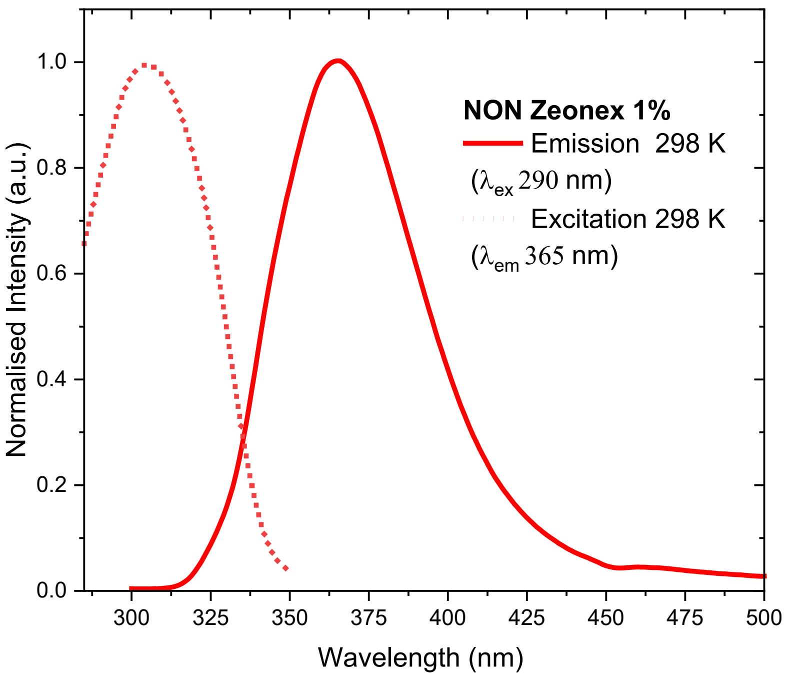

Figure S19: Excitation and Emission spectra for NON in a Zeonex matrix (1 wpc) at 298 K

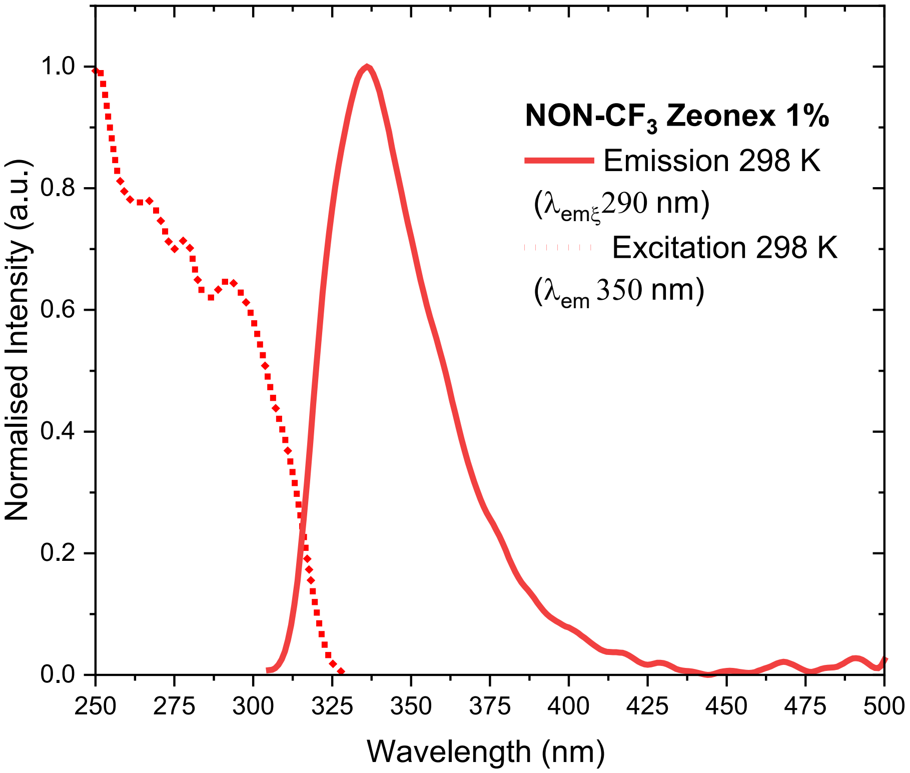

Figure S20: Excitation and Emission spectra for NON-CF_3 in a Zeonex matrix (1 wpc) at 298 K

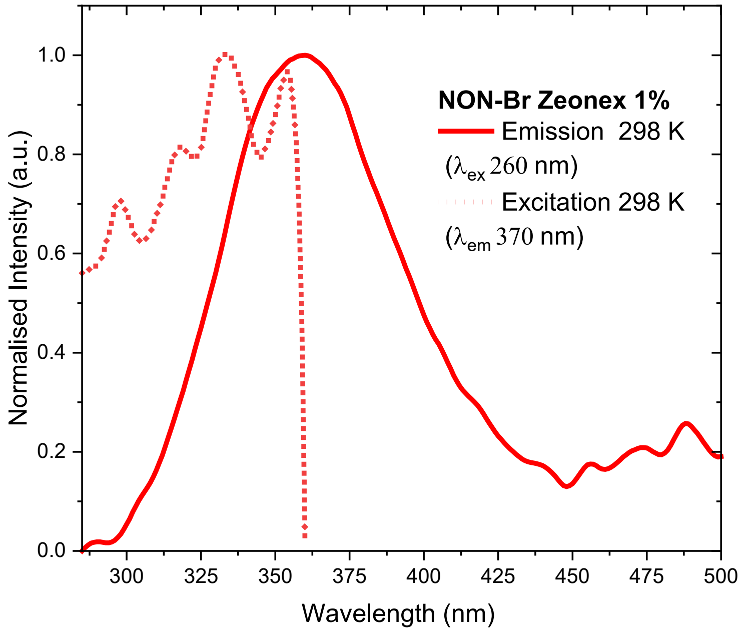

Figure S21: Excitation and Emission spectra for NON-Br in a Zeonex matrix (1% by weight) at 298 K.

a.

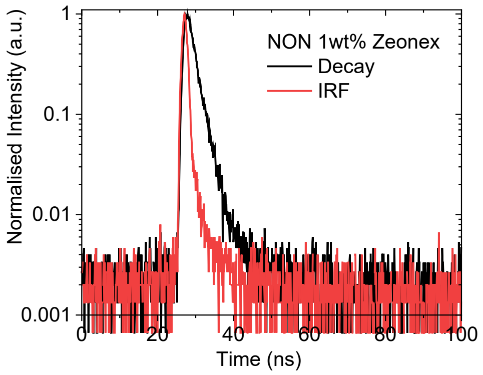

b.

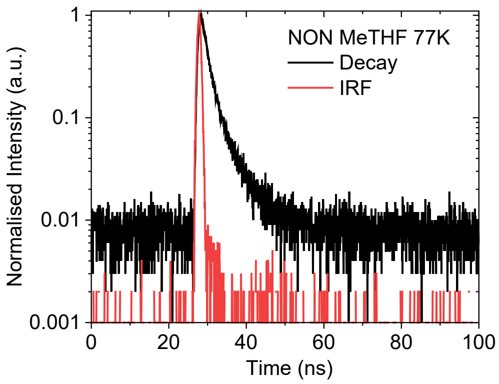

c.

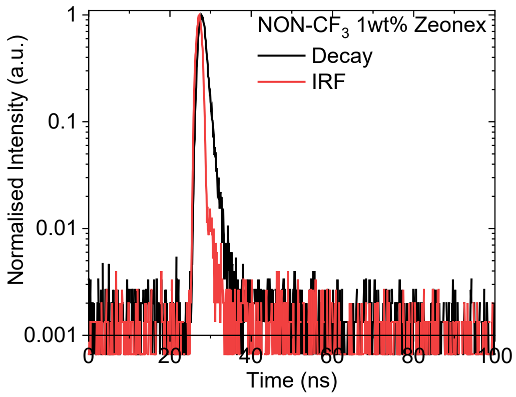

d.

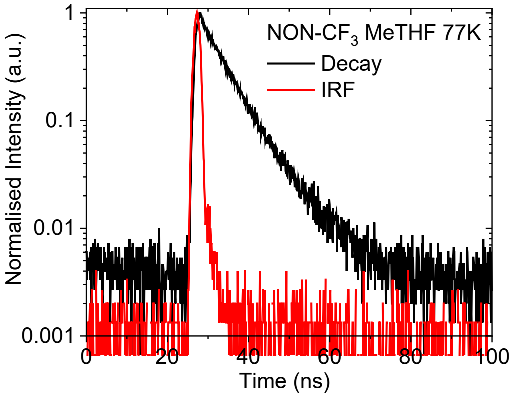

e.

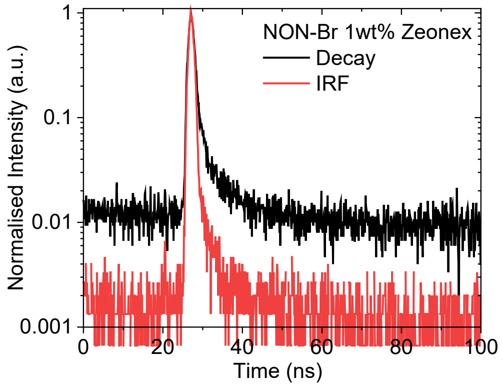

f.

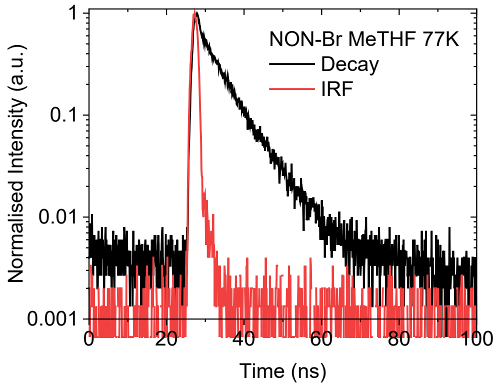

Figure S22. Excited state lifetime traces measured by Time Correlated Single Photon Counting (TSCPC, black) and Instrument Response Function (red) in methylcyclohexane (MCH) solution for compound **NON** at 295 K in 1 wt% Zeonex film (a) and at 77 K MeTHF glass (b); **NON-CF$_3$** at 295K in 1% wt% Zeonex film (c) and at 77 K MeTHF glass (d); in 1 wt% Zeonex films for **NON-Br** at 295K (e) and at 77K MeTHF glass (f).

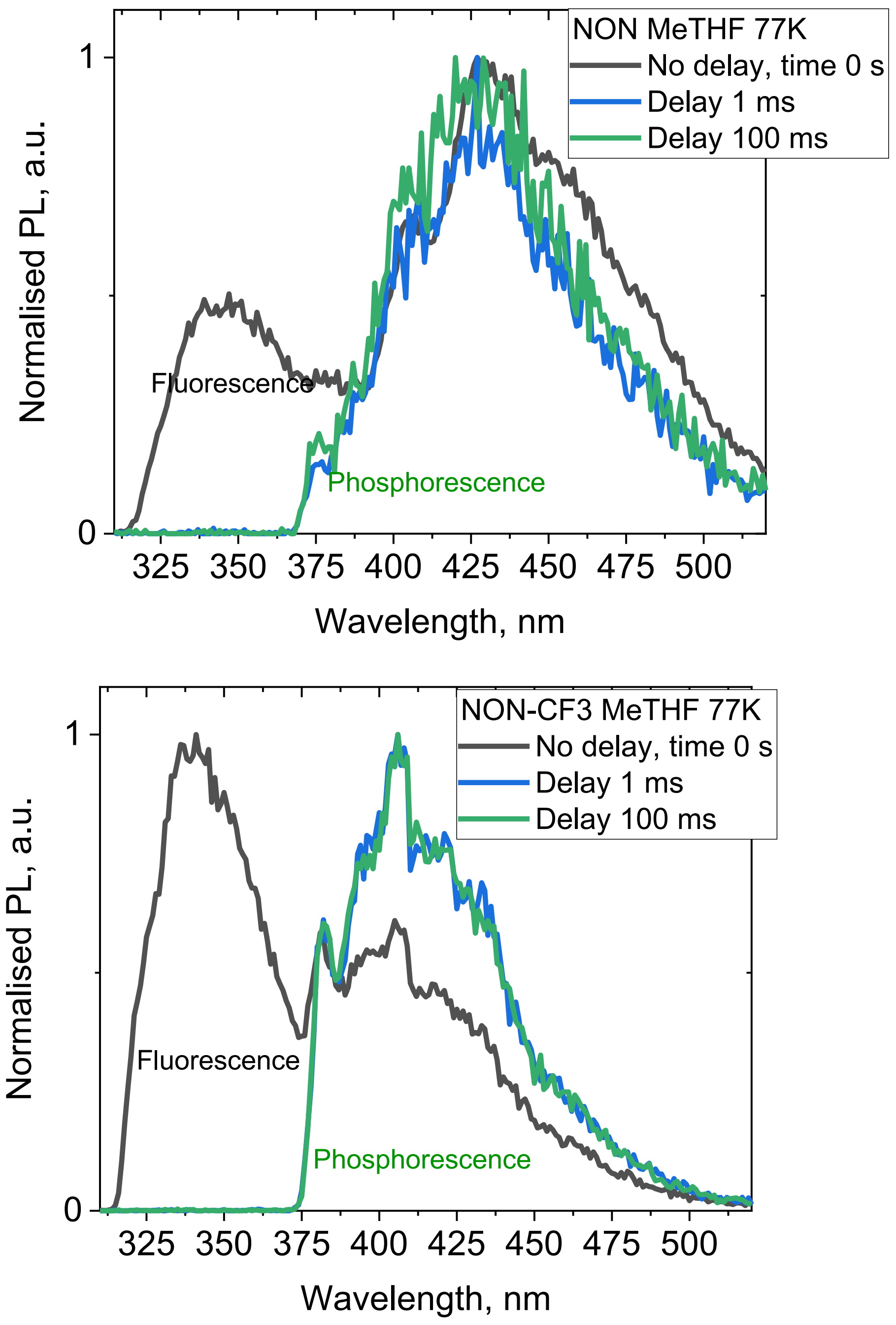

Figure S23. Steady state and PL profiles after various delays at 77 K for MeTHF frozen glass of compounds NON (a) and NON-CF3 (b) showing clear fluorescence and phosphorescence contribution.

6. Device fabrication and characterisation

For the fabrication of OLED devices, ITO coated substrates (~15 Ω/cm^2) were cleaned with acetone and isopropyl alcohol, and then O_2 plasma treatment was applied to align the energy level with a hole transporting layer. All layers, including organic layers and a LiF/aluminium cathode, were thermally deposited in high vacuum (~10^{-7} torr).

The performance of the OLED devices was measured by a Keithley 2635 source-meter and a calibrated Si photodiode. The EL spectra were recorded by an Ocean Optics Flame spectrometer. The voltage pulse was given by a Keithley 2401 function generator (20,000 Hz frequency and 10 μs pulse width).

7. Computational results

Table S4. Optimised gas phase structures and molecular orbital distribution of the HOMO (middle) and LUMO (right) for NON-materials with viewing from the front (top), side (middle) and top (bottom panel).

	HOMO	LUMO

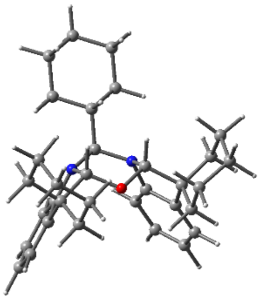<br>NON<br>Overlap integral: 0.62	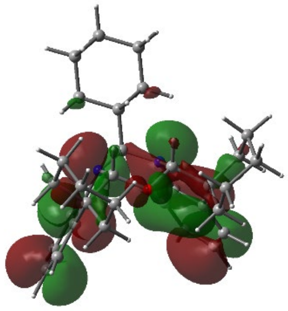<br>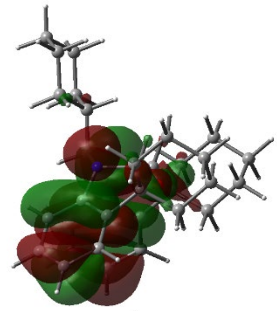<br>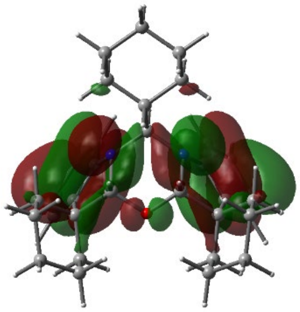	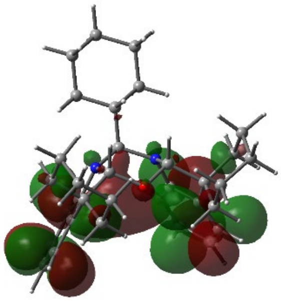<br>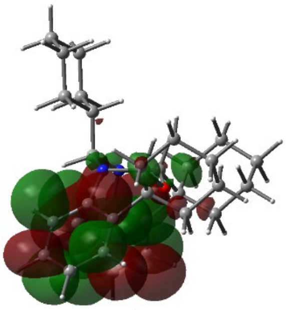<br>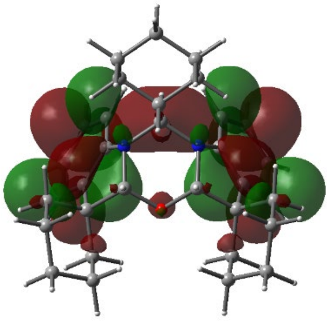

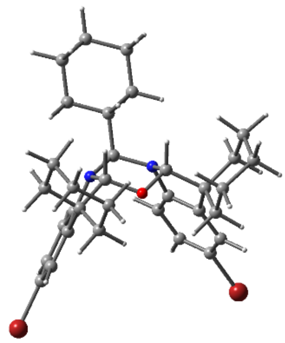<br>NON-Br<br>Overlap integral: 0.62	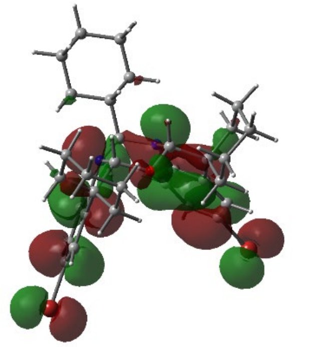	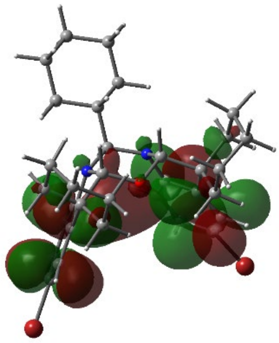
	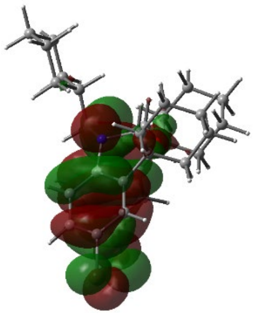	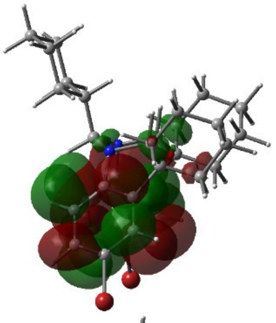
	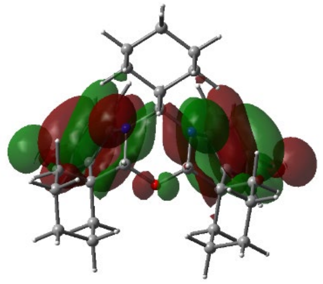	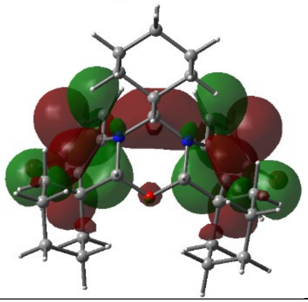

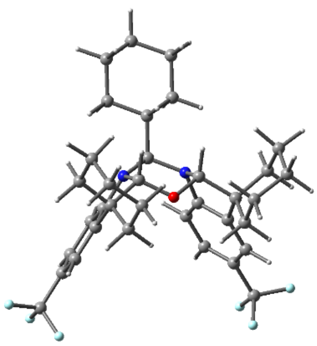 NON-CF_3 Overlap integral: 0.66	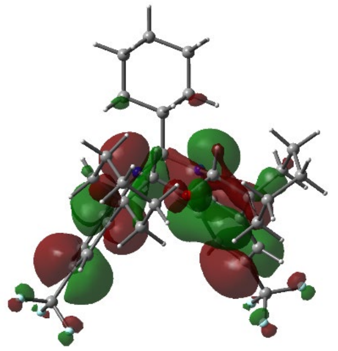 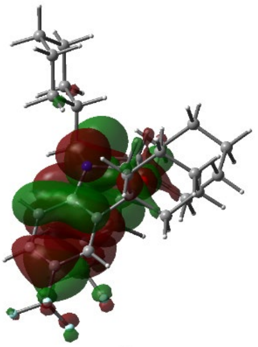 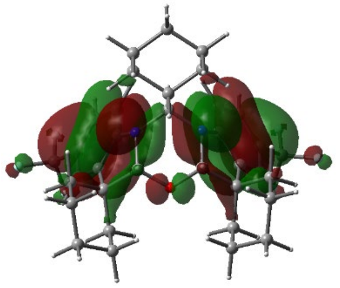	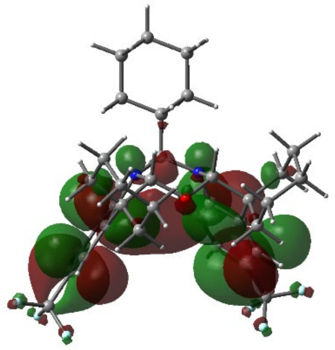 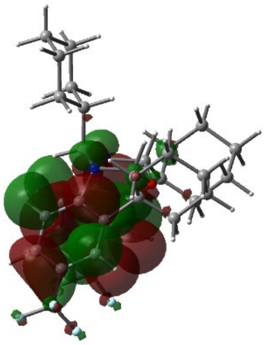 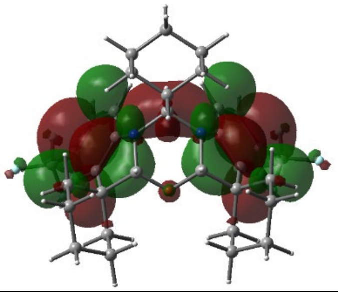

Table S5. Dipole moments of S_0 and S_1 states in S_0 geometry.

	S_0	$S_1@S_0$
NON	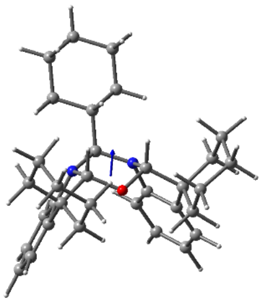 2.1D	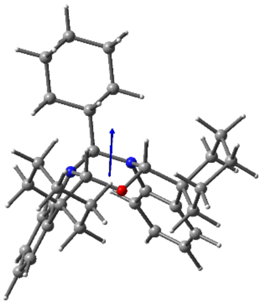 3.7D

NON-Br	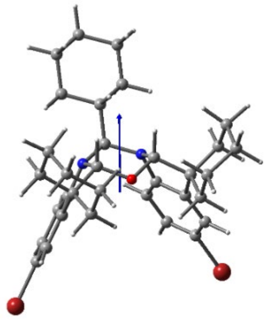 5.9D	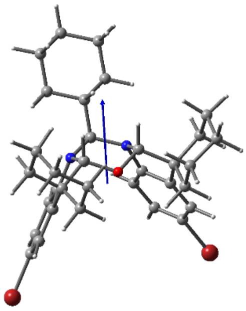 6.7D
NON-CF_3	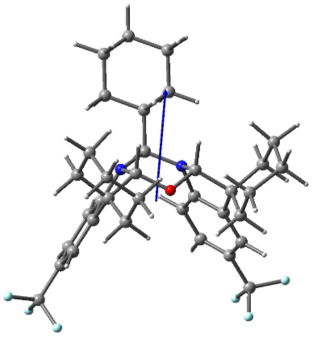 7.9D	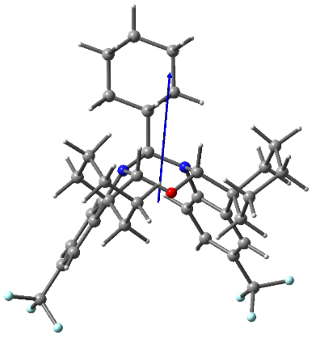 9.4D

Table S6. Theoretically calculated S_1 and T_1 energy levels, orbital (HOMO, LUMO) contributions to vertical excitations ($S_0 \rightarrow S_n$, $S_0 \rightarrow T_n$, n = 1, 2, 3, 4, 5) and oscillator strength coefficients.

	Excitation energy	Character	Oscillator strength
NON	S_1: 4.63eV = 268nm	HOMO – LUMO (81%)	0.000
	S_2: 4.87eV = 255nm	HOMO – LUMO+1 (57%)<br>HOMO-1 – LUMO (25%)	0.0876
	S_3: 5.21eV = 238nm	HOMO – LUMO+2 (88%)	0.1708
	S_4: 5.59eV = 222nm	HOMO-1 – LUMO (62%)<br>HOMO – LUMO+1 (35%)	0.0040
	S_5: 5.65eV = 219nm	HOMO – LUMO+3 (68%)	0.1289
	T_1: 3.89eV = 318nm	HOMO – LUMO+2 (57%)<br>HOMO-1 – LUMO+3 (23%)	
	T_2: 3.92eV = 316nm	HOMO – LUMO (72%)<br>HOMO-1 – LUMO+1 (24%)	
	T_3: 3.96eV = 313nm	HOMO – LUMO+3 (29%)<br>HOMO-1 – LUMO+2 (29%)	
	T_4: 4.04eV = 307nm	HOMO – LUMO+1 (40%)<br>HOMO-1 – LUMO (33%)	
	T_5: 4.57eV = 271nm	HOMO-2 – LUMO (49%)<br>HOMO-3 – LUMO+1 (32%)	
NON-Br	S_1: 4.43eV = 280nm	HOMO – LUMO (82%)	0.0003
	S_2: 4.66eV = 266nm	HOMO – LUMO+1 (57%)<br>HOMO-1 – LUMO (28%)	0.0852

	S_3: 5.00eV = 248nm	HOMO – LUMO+2 (88%)	0.2610
	S_4: 5.28eV = 235nm	HOMO – LUMO+5 (58%)<br>HOMO-1 – LUMO+6 (29%)	0.0016
	S_5: 5.30eV = 234nm	HOMO – LUMO+6 (40%)<br>HOMO-1 – LUMO+5 (24%)	0.0007
	T_1: 3.72eV = 333nm	HOMO – LUMO (69%)<br>HOMO-1 – LUMO+1 (20%)	
	T_2: 3.75eV = 331nm	HOMO – LUMO+2 (57%)<br>HOMO-1 – LUMO+3 (22%)	
	T_3: 3.81eV = 326nm	HOMO – LUMO+1 (34%)<br>HOMO-1 – LUMO (19%)	
	T_4: 3.85eV = 322nm	HOMO – LUMO+3 (28%)<br>HOMO-1 – LUMO (21%)<br>HOMO – LUMO+1 (21%)<br>HOMO-1 – LUMO+2 (20%)	
	T_5: 4.54eV = 273nm	HOMO-2 – LUMO (51%)<br>HOMO-3 – LUMO+1 (34%)	
NON-CF_3	S_1: 4.63eV = 268nm	HOMO – LUMO (75%)	0.0066
	S_2: 4.86eV = 255nm	HOMO – LUMO+2 (50%)<br>HOMO-1 – LUMO (22%)	
	S_3: 5.04eV = 246nm	HOMO – LUMO+1 (78%)	
	S_4: 5.48eV = 226nm	HOMO – LUMO+3 (70%)	
	S_5: 5.59eV = 222nm	HOMO-1 – LUMO (64%)<br>HOMO – LUMO+2 (31%)	
	T_1: 3.76eV = 329nm	HOMO – LUMO (33%)<br>HOMO – LUMO+1 (32%)<br>HOMO-1 – LUMO+3 (19%)	
	T_2: 3.87eV = 321nm	HOMO – LUMO+3 (40%)<br>HOMO-1 – LUMO+1 (26%)	
	T_3: 3.99eV = 311nm	HOMO – LUMO (40%)<br>HOMO – LUMO+1 (28%)<br>HOMO-1 – LUMO+2 (19%)	
	T_4: 4.07eV = 304nm	HOMO – LUMO+2 (49%)<br>HOMO-1 – LUMO (27%)	
	T_5: 4.55eV = 272nm	HOMO-2 – LUMO (46%)<br>HOMO-3 – LUMO+2 (34%)	

Table S7. Natural transition orbitals for vertical excited states.

		HONTO	LUNTO
NON	S_1	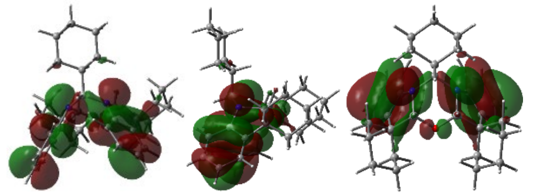	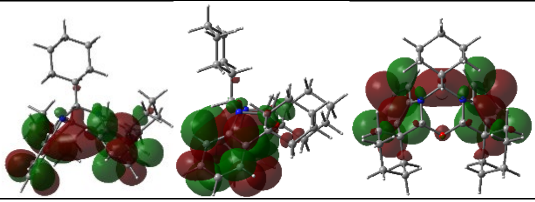

	S_2	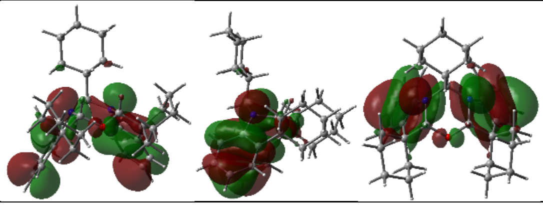	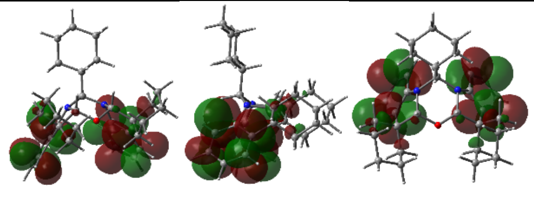
	S_3	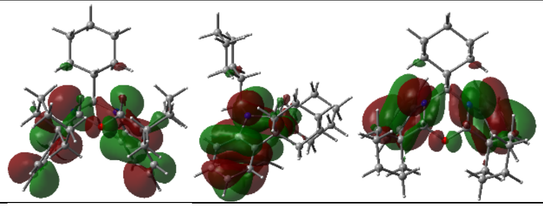	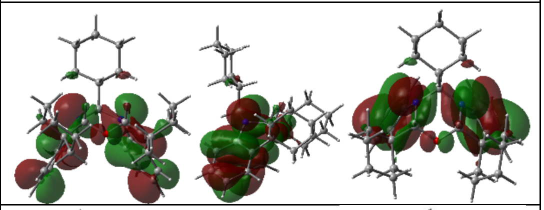
	S_4	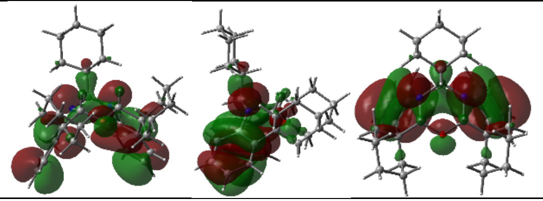	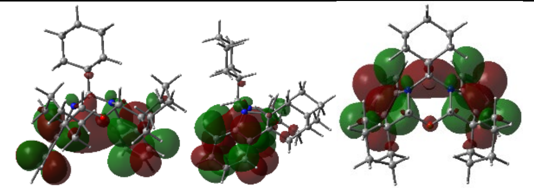
	S_5	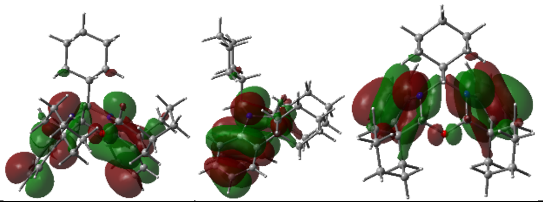	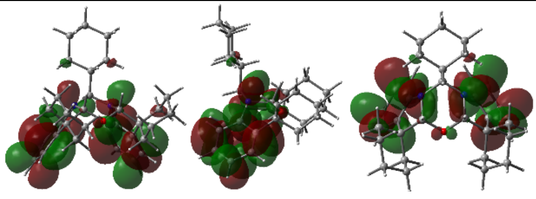
	T_1	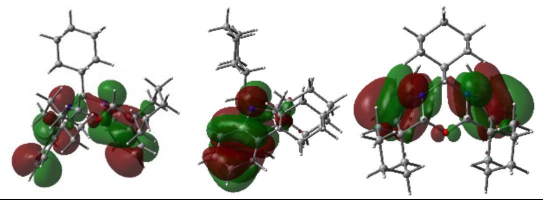	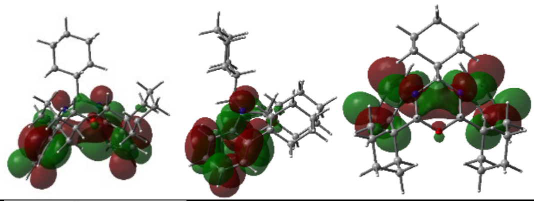
	T_2	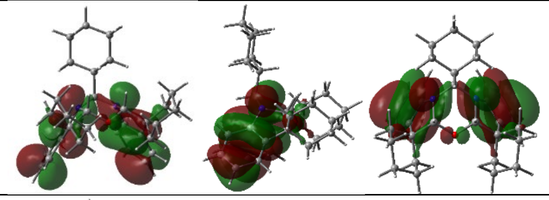	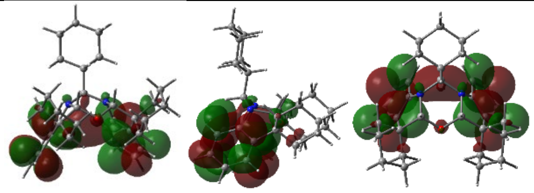
	T_3	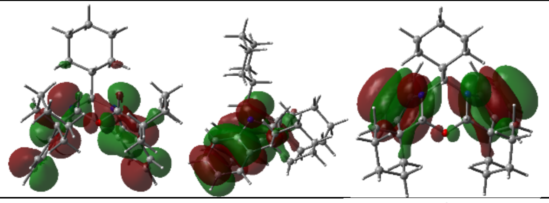	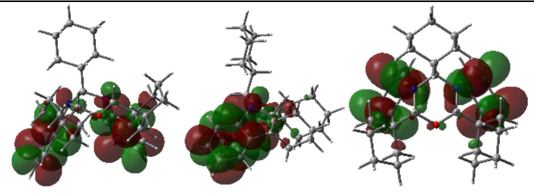
	T_4	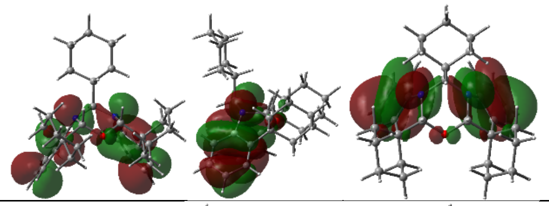	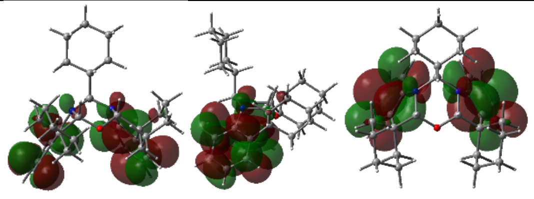
	T_5	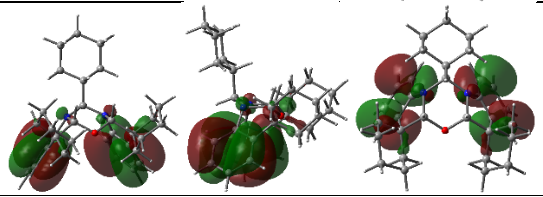	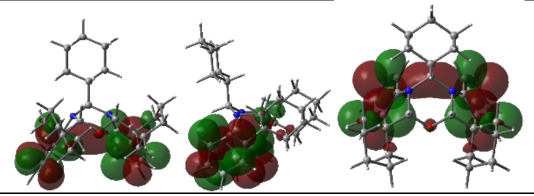

NON-Br	S_1	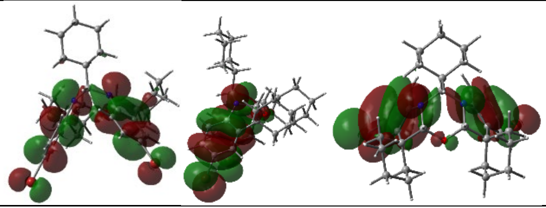	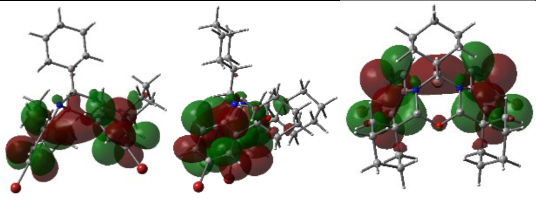
	S_2	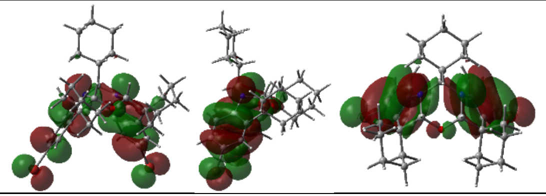	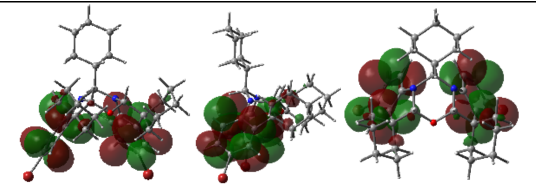
	S_3	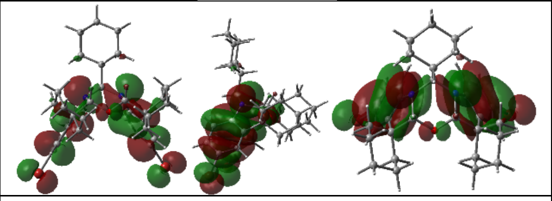	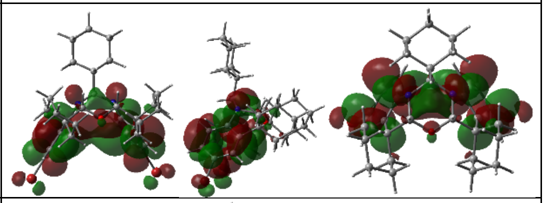
	S_4	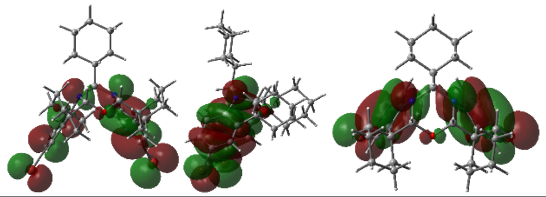	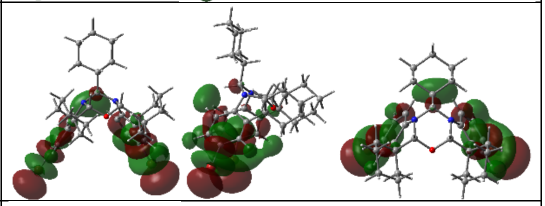
	S_5	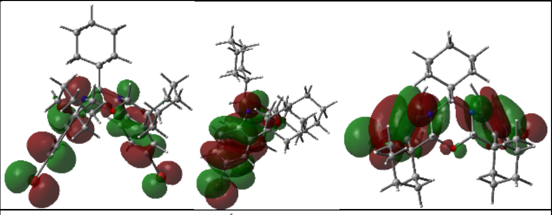	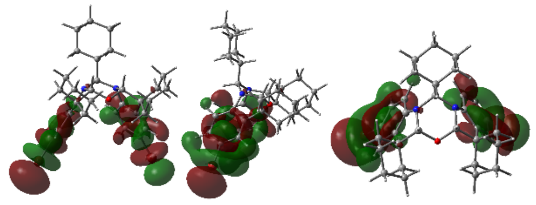
	T_1	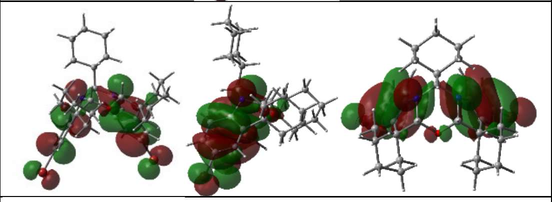	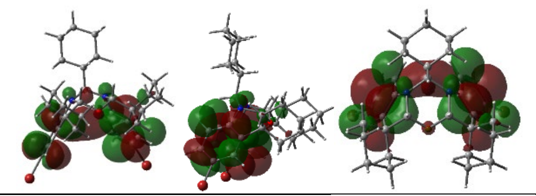
	T_2	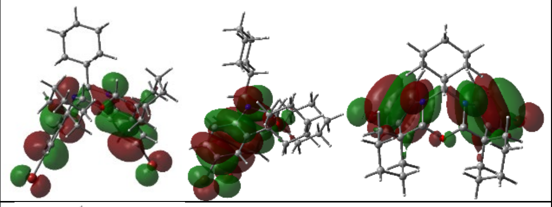	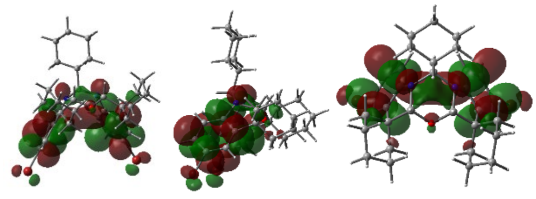
	T_3	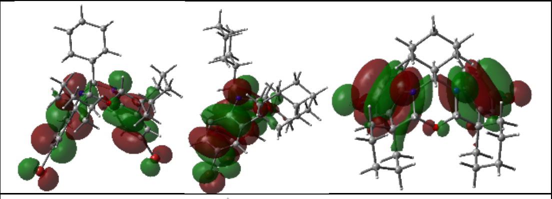	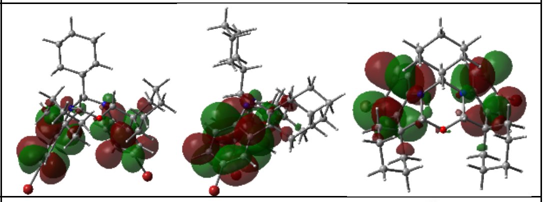
	T_4	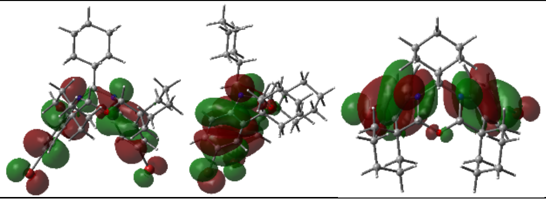	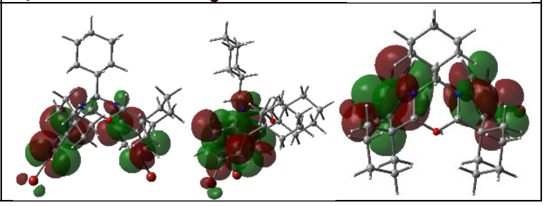

	T_5	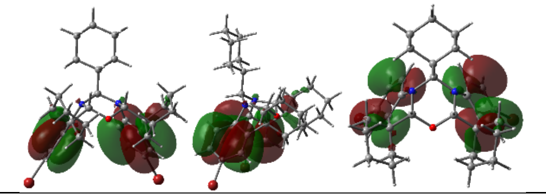	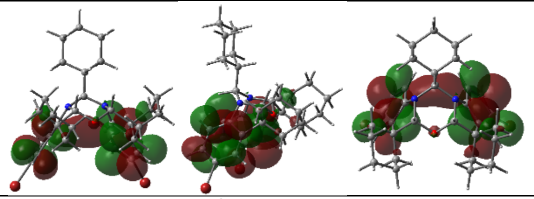
NON-CF_3	S_1	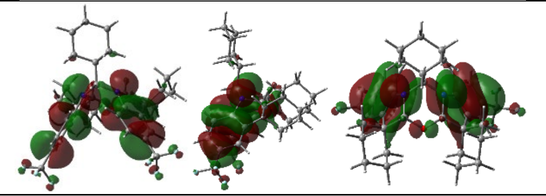	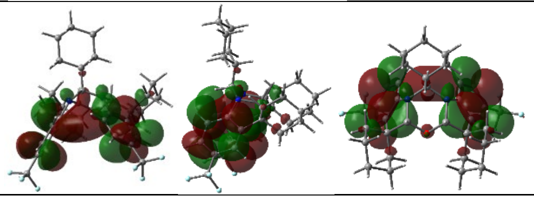
	S_2	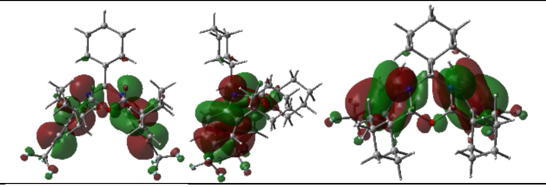	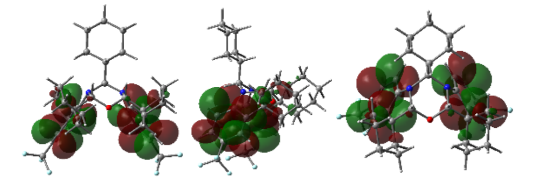
	S_3	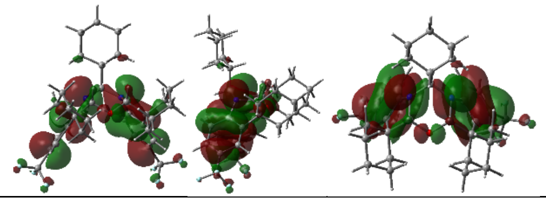	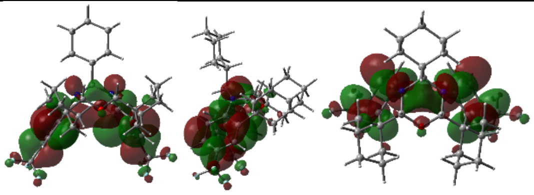
	S_4	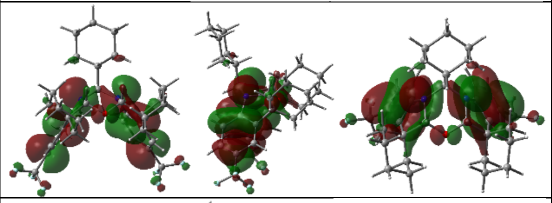	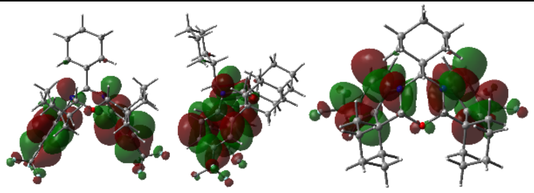
	S_5	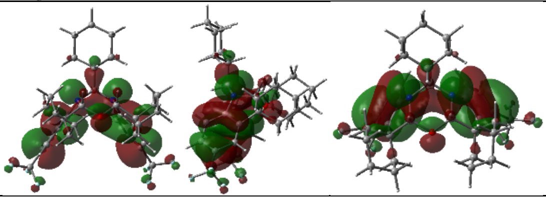	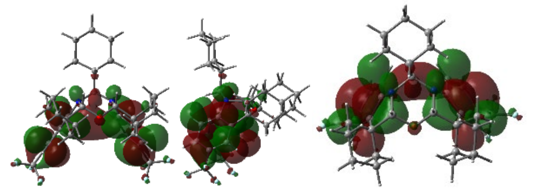
	T_1	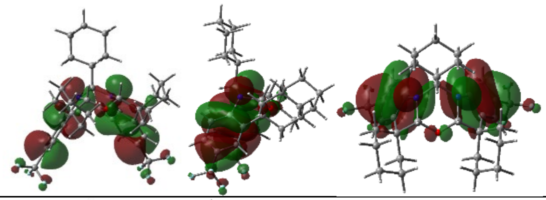	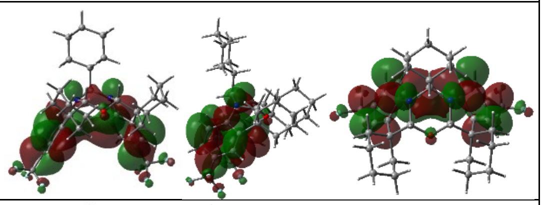
	T_2	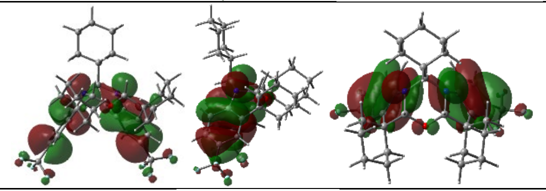	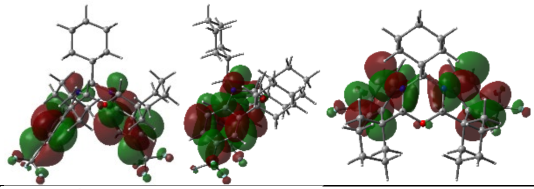
	T_3	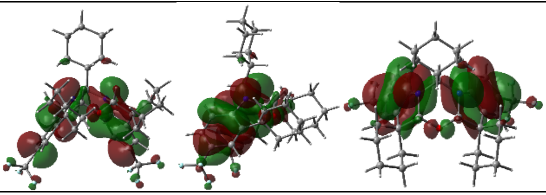	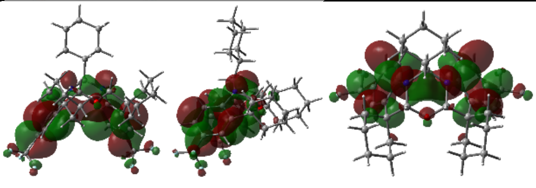

	T_4	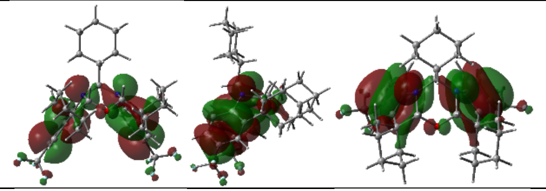	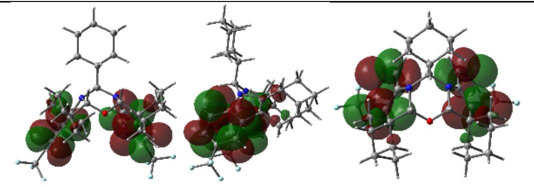
	T_5	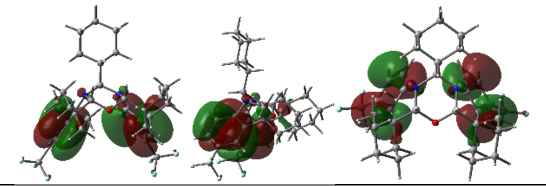	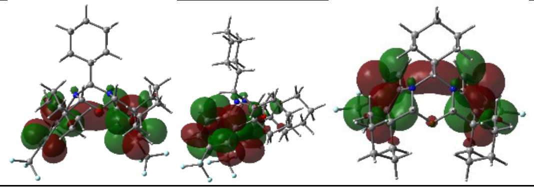

Table S8. Summary of the fluorescence lifetime fitting results for NON-materials in MeTHF solution at 298 and 77 K.

Compound	x_1	τ_1[ns]	x_2	τ_2[ns]	$\langle\tau\rangle_I$	χ_r^2
298 K						
NON	1.00	1.394			1.4	1.03
NON-CF_3	1.00	0.8803			0.9	0.87
NON-Br	0.82	0.4221	0.19	4.326	1.1	1.29
77 K						
NON	1.00	2743				1.15
NON-CF_3	0.08	0.3819	0.92	6.4477	5.9	1.27

*Emission from NON-Br at 315nm was too weak at 77 K measurement.

Table S9. Summary of the lifetime fitting results for NON-materials in 1 wt% Zeonex matrix at 298 K.

8. Optimised coordinates:

Compound	x_1	τ_1[ns]	x_2	τ_2	$\langle\tau\rangle_I$	χ_r^2
298 K						
NON	1.00	1.8055			1.8	1.31
NON-CF_3	1.00	0.8803			0.9	0.87
NON-Br	0.83	0.3604	0.17	4.7394	1.1	1.54

78

NON

O	0.00015100	-1.06562100	-0.54311200
N	1.19578600	0.69664000	0.45947800
N	-1.19585400	0.69655300	0.45917100
C	-0.00003300	2.65449700	-0.43793400
H	-0.00013400	2.22554400	-1.45096600
C	-2.40512600	-1.15369000	-0.37446600
C	-1.25205600	3.51211500	-0.28481600
H	-2.14418000	2.89532100	-0.41383300
H	-1.27951300	3.89939800	0.74407100
C	2.40536700	-1.15352000	-0.37387600
C	-1.81175200	0.07930500	1.55761100
C	0.00005400	5.53410000	-1.08968800
H	0.00013600	5.96991200	-0.08304300
H	0.00005300	6.36613800	-1.79755200
C	3.62037400	-0.61113400	-2.55897900

H	2.80583100	-0.00545600	-2.97213500
H	4.54621500	-0.19452900	-2.96260900
C	1.82160200	0.46143600	2.89041500
H	1.27264200	1.32749700	3.23926000
C	2.53442400	-1.03443600	1.12626500
C	1.81125200	0.07925800	1.55810900
C	-1.25000900	4.68070200	-1.26240100
H	-1.27581900	4.28919100	-2.28690100
H	-2.15142200	5.28352600	-1.13158800
C	-1.16543300	-0.27336000	-0.61897600
H	-1.17842600	0.24024900	-1.59004400
C	-3.63365700	-0.50571500	-1.03813900
H	-4.51958200	-1.02053800	-0.64831900
H	-3.70353500	0.53825100	-0.71430400
C	-1.82266200	0.46166200	2.88986500
H	-1.27388500	1.32780000	3.23881000
C	1.16568800	-0.27326300	-0.61870200
H	1.17887400	0.24037600	-1.58975200
C	3.63407200	-0.50537800	-1.03705900
H	3.70377800	0.53854900	-0.71306700
H	4.51990500	-1.02019800	-0.64702700
C	-0.00008400	1.51215700	0.58305200
H	-0.00021600	1.96786700	1.57721100
C	1.25001100	4.68058200	-1.26256000
H	1.27565300	4.28907700	-2.28706800
H	2.15149800	5.28332200	-1.13186200
C	3.27211500	-1.78266200	2.01935200
H	3.83438200	-2.64635500	1.68043400
C	-2.53473400	-1.03445400	1.12561500
C	1.25209800	3.51197800	-0.28499100
H	1.27977200	3.89922300	0.74390300
H	2.14414300	2.89510500	-0.41416900
C	-3.27277400	-1.78257800	2.01849900
H	-3.83490100	-2.64631500	1.67945900
C	2.23987600	-2.58483600	-0.87379900
H	3.11175100	-3.15674200	-0.53210300
H	1.35625200	-3.03223400	-0.41645400
C	-2.23934100	-2.58505000	-0.87416800
H	-3.11125700	-3.15700000	-0.53265200
H	-1.35580500	-3.03229900	-0.41650700
C	2.17472100	-2.64844200	-2.39482200
H	1.29804000	-2.08683100	-2.74162800
H	2.03599200	-3.68141600	-2.72154600
C	-3.28319700	-1.41707100	3.36642400
H	-3.84817400	-1.99908100	4.08283400
C	-3.61948300	-0.61167600	-2.56004100
H	-4.54522100	-0.19518200	-2.96401700
H	-2.80484500	-0.00601300	-2.97303100
C	3.28198800	-1.41732200	3.36732600
H	3.84668900	-1.99941000	4.08389200

C	2.56429900	-0.30576300	3.78774600
H	2.57591600	-0.02663600	4.83413300
C	-2.17373200	-2.64885000	-2.39516300
H	-1.29699100	-2.08722200	-2.74178700
H	-2.03483000	-3.68185900	-2.72170500
C	3.43830800	-2.05593600	-3.00745800
H	4.30233000	-2.64618100	-2.67798200
H	3.40742700	-2.11632900	-4.09773500
C	-2.56570600	-0.30543800	3.78699100
H	-2.57776000	-0.02617300	4.83333600
C	-3.43718500	-2.05653000	-3.00825700
H	-3.40597700	-2.11708200	-4.09851500
H	-4.30126000	-2.64678900	-2.67894800

78
NON_Br

Br	-4.55233900	3.77763400	-0.34399300
Br	3.89482800	4.43509500	-0.34807000
O	0.08664900	-0.95515400	1.27825500
C	-2.17232100	-0.55743800	2.75061000
H	-1.33535300	0.13240300	2.86710500
H	-3.08220400	-0.02325300	3.05136200
N	1.27003700	-0.97344000	-0.75415000
C	2.29629000	-0.26435100	2.72493800
H	3.12097900	0.39748900	3.01738700
H	1.36664100	0.28650300	2.87456100
N	-1.11336100	-1.15387100	-0.73745500
C	-3.44891800	2.25756900	-0.52944500
C	3.12604400	1.83331100	0.49904900
H	3.68040700	2.06807800	1.40019400
C	-1.91173000	0.95808500	-1.85809700
H	-1.36621600	0.82126200	-2.78339600
C	1.77603000	0.33121300	-0.82487600
C	2.48543800	0.62331100	0.34235700
C	0.36342800	-4.97329100	-3.97601000
H	0.33207800	-4.36046700	-4.88515500
H	0.43546800	-6.01515200	-4.29596800
C	3.04155800	2.76207200	-0.53683600
C	1.48513000	-3.12853600	-2.69374400
H	1.48140800	-2.46927100	-3.57387000
H	2.35449900	-2.84917200	-2.09431500
C	2.46849900	-0.58752300	1.24460800
C	3.86512600	-2.61897400	1.92348100
H	3.10581000	-3.35589200	1.63812900
H	4.83267900	-3.10269700	1.77160300
C	-2.54826800	0.24524400	0.36347400
C	3.76565700	-1.38773400	1.02809000
H	3.84475500	-1.65971700	-0.02982100
H	4.60120300	-0.71307300	1.24766100
C	-1.82022100	0.05289500	-0.81261200
C	-2.73552800	2.07210200	-1.70205800

H	-2.81957600	2.79993300	-2.49825400
C	0.09915900	-1.40359500	-1.49885000
H	0.04920800	-0.81889700	-2.42184300
C	-1.04609300	-1.52927500	0.66329200
H	-0.98369500	-2.62146600	0.75890400
C	-2.01015700	-1.78848700	3.63337700
H	-1.09535800	-2.32153900	3.34460900
H	-1.88332500	-1.48755200	4.67544900
C	-2.32560200	-0.93363900	1.28051800
C	3.67076600	-2.24924800	3.38875400
H	3.72083900	-3.14118000	4.01714700
H	4.48761100	-1.58875600	3.70435900
C	1.28026400	-1.36096400	0.64426800
H	1.37668500	-2.45122400	0.73409400
C	-3.37919600	-3.13947800	2.03004600
H	-2.52175000	-3.75740100	1.74031400
H	-4.26391900	-3.76888000	1.91031400
C	2.34475400	2.47968700	-1.69999300
H	2.29799500	3.22086700	-2.48684900
C	-3.36909800	1.34144700	0.51797200
H	-3.93927000	1.49854300	1.42613600
C	-3.21287600	-2.71266000	3.48319200
H	-4.11389600	-2.17684500	3.80598300
H	-3.11369000	-3.58802900	4.12877800
C	-1.01471300	-3.29305100	-2.72037600
H	-1.92555500	-3.12821600	-2.14055600
H	-1.07605300	-2.63948400	-3.60267600
C	0.20482500	-2.87721400	-1.90386700
H	0.23541300	-3.49503500	-0.99428300
C	-0.91255300	-4.74322300	-3.17645700
H	-1.79212800	-5.01638600	-3.76336700
H	-0.90412000	-5.39565200	-2.29460600
C	-3.48898800	-1.92752000	1.10987500
H	-4.41374500	-1.38447200	1.33667400
H	-3.54703800	-2.23148800	0.05924100
C	1.70389000	1.25144200	-1.85837700
H	1.16840000	1.04153700	-2.77574400
C	2.34585100	-1.52178900	3.58448200
H	2.20012000	-1.26256300	4.63524000
H	1.51614600	-2.18325700	3.30434900
C	1.58179200	-4.57883400	-3.15122000
H	1.64083000	-5.22827000	-2.26905800
H	2.50125900	-4.73377700	-3.71981700

84

NON_CF3

F	3.76423800	4.48774400	-0.67287100
F	4.06693100	4.59353400	1.45911100
F	5.44341300	3.49344800	0.21760300
O	-0.00524500	-0.88565900	-1.27891000
F	-3.71818900	4.52317400	-0.66846500

F	-4.02968200	4.62617100	1.46230700
F	-5.40980100	3.54087100	0.21171400
N	1.18800500	-1.00340900	0.74275700
N	-1.19564800	-0.99362800	0.74475400
C	3.30424800	1.61291400	-0.47921800
H	3.87547000	1.80604000	-1.38137400
C	2.55316800	0.47115100	-0.34223400
C	3.32167200	2.53847800	0.57014300
C	-0.00997300	-2.81548600	1.91048400
H	-0.01078700	-3.43761600	1.00347400
C	1.15796400	-1.38668200	-0.65764900
H	1.16410200	-2.48089500	-0.74954800
C	1.84291500	1.14318600	1.87707800
H	1.29102700	0.97189900	2.79242200
C	2.40473400	-0.71555200	-1.26335900
C	2.60206300	2.30286700	1.73023600
H	2.62922700	3.03076800	2.52986000
C	-1.17103000	-1.37794600	-0.65542500
H	-1.18547200	-2.47214700	-0.74668600
C	-2.54949700	0.49182900	-0.33958200
C	1.82267900	0.23598600	0.82932800
C	-1.81900600	0.25161800	0.83097500
C	-3.29073800	1.63998200	-0.47665200
H	-3.86203100	1.83690500	-1.37794100
C	-0.00461700	-1.33988400	1.50132200
H	-0.00147100	-0.75075200	2.42238000
C	4.14292700	3.77887800	0.40246000
C	-1.82919900	1.16032200	1.87754700
H	-1.27706400	0.98537400	2.79204700
C	-2.41361600	-0.69754500	-1.25916800
C	-2.24969100	-0.32994100	-2.73044200
H	-3.12429400	0.26480800	-3.02161400
H	-1.36938900	0.30225100	-2.85474200
C	3.62176700	-1.64266900	-1.08793600
H	4.51544000	-1.04672800	-1.30583900
H	3.69043600	-1.94718300	-0.03796000
C	-3.63804400	-1.61386800	-1.07856100
H	-3.70655100	-1.91552600	-0.02775900
H	-4.52713100	-1.01072600	-1.29540600
C	2.24000700	-0.34352600	-2.73343900
H	1.36493700	0.29659500	-2.85400900
H	3.11897300	0.24419300	-3.02574200
C	3.58782000	-2.85514000	-2.01305100
H	4.50593600	-3.43353800	-1.88823800
H	2.76490500	-3.52237100	-1.73243000
C	-3.29806600	2.56695200	0.57157700
C	-2.57834000	2.32649800	1.73060300
H	-2.59760600	3.05561100	2.52934900
C	-4.10785500	3.81479100	0.40328900
C	2.15603000	-1.57862500	-3.62133700

H	1.27198500	-2.16521500	-3.34088100
H	2.01873900	-1.28137900	-4.66304000
C	1.24125300	-3.14856800	2.71692600
H	1.27148700	-2.48525700	3.59352900
H	2.13472000	-2.93300800	2.12652300
C	-2.17889100	-1.56760700	-3.61599700
H	-2.04192400	-1.27370000	-4.65869000
H	-1.29915400	-2.16125500	-3.33682500
C	-3.61695700	-2.82854800	-2.00112900
H	-2.79894200	-3.50211800	-1.72134600
H	-4.53958800	-3.39888400	-1.87256100
C	3.40865700	-2.43282600	-3.46592300
H	3.36397500	-3.30992900	-4.11509300
H	4.28004300	-1.84563800	-3.78026600
C	-1.26638200	-4.59127800	3.18201500
H	-2.16907400	-4.79897100	3.76039400
H	-1.29257800	-5.24969700	2.30503700
C	-1.26482300	-3.14116700	2.71423000
H	-1.29342900	-2.47737000	3.59050500
H	-2.15578400	-2.92080100	2.12179400
C	-3.43829500	-2.41076200	-3.45536100
H	-3.40285800	-3.28952700	-4.10285700
H	-4.30553800	-1.81684300	-3.76851600
C	1.23354600	-4.59871600	3.18445700
H	1.25758800	-5.25721600	2.30748800
H	2.13386300	-4.81172100	3.76459900
C	-0.01812600	-4.90154900	3.99803100
H	-0.02158600	-5.94313300	4.32647300
H	-0.01713300	-4.28065200	4.90218000

9. Supplementary References

1. Gritzner, G.; Kůta, J. Recommendations on reporting electrodepotentials in nonaqueous solvents: IUPC commission on electro-chemistry. *Electrochim. Acta*, **1984**, 29, 869−873.

2. *Programs CrysAlisPro, Oxford Diffraction Ltd., Abingdon, UK (2010).*

3. Sheldrick, G. M. Crystal structure refinement with SHELXL. *Acta Crystallogr. Sect. C* **2015**, 71, 3–8.

4. Dolomanov, O. V.; Bourhis, L. J.; Gildea, R. J.; Howard, J. A. K.; Puschmann, H. OLEX2: a complete structure solution, refinement and analysis program. *J. Appl. Crystallogr.* **2009**, *42*, 339–341.
