# Supplementary material for: High triplet energy host material with a 1,3,5-oxadiazine core from a one-step interrupted Fischer indolization
Source: Commun Chem. 2024 Dec 19;7:298. doi: 10.1038/s42004-024-01377-y (PMC11659277; doi:10.1038/s42004-024-01377-y)
Supplement: Supplementary file 3 — Description of Additional Supplementary Files [file 42004_2024_1377_MOESM3_ESM.pdf]

Description of Additional Supplementary files

File name: Supplementary Data 1

File description: The combined CIF file contains crystallographic data for the title materials.
